# Comparative Proteomic Analysis of Differentially Expressed Proteins Induced by Hydrogen Sulfide in *Spinacia oleracea* Leaves

**DOI:** 10.1371/journal.pone.0105400

**Published:** 2014-09-02

**Authors:** Juan Chen, Ting-Wu Liu, Wen-Jun Hu, Martin Simon, Wen-Hua Wang, Juan Chen, Xiang Liu, Hai-Lei Zheng

**Affiliations:** 1 State Key Laboratory of Soil Erosion and Dryland Farming on the Loess Plateau, Northwest A&F University, Yangling, Shanxi, P.R. China; 2 Key Laboratory for Subtropical Wetland Ecosystem Research of MOE, College of the Environment and Ecology, Xiamen University, Xiamen, Fujian, P.R. China; 3 Department of Biology, Huaiyin Normal University, Huaian, Jiangsu, P.R. China; University of Virginia, United States of America

## Abstract

Hydrogen sulfide (H_2_S), as a potential gaseous messenger molecule, has been suggested to play important roles in a wide range of physiological processes in plants. The aim of present study was to investigate which set of proteins is involved in H_2_S-regulated metabolism or signaling pathways. *Spinacia oleracea* seedlings were treated with 100 µM NaHS, a donor of H_2_S. Changes in protein expression profiles were analyzed by 2-D gel electrophoresis coupled with MALDI-TOF MS. Over 1000 protein spots were reproducibly resolved, of which the abundance of 92 spots was changed by at least 2-fold (sixty-five were up-regulated, whereas 27 were down-regulated). These proteins were functionally divided into 9 groups, including energy production and photosynthesis, cell rescue, development and cell defense, substance metabolism, protein synthesis and folding, cellular signal transduction. Further, we found that these proteins were mainly localized in cell wall, plasma membrane, chloroplast, mitochondria, nucleus, peroxisome and cytosol. Our results demonstrate that H_2_S is involved in various cellular and physiological activities and has a distinct influence on photosynthesis, cell defense and cellular signal transduction in *S. oleracea* leaves. These findings provide new insights into proteomic responses in plants under physiological levels of H_2_S.

## Introduction

Hydrogen sulfide (H_2_S) has been emerging as a potential messenger molecule, strikingly similar to nitric oxide (NO) and carbon monoxide (CO), involved in the modulation of a wide range of physiological processes in animals and plants [1]–[6]. Since the 1970s, the phenomenon of H_2_S emission from plants has been demonstrated by many researchers [7]–[9]. Moreover, the production of H_2_S can be altered under biotic or abiotic stresses [10]. In addition, H_2_S has a dual function, either as a cytotoxin or a cytoprotectant, which depends on the concentration of H_2_S and the status of the environment. At low concentration, H_2_S has an obvious signaling regulatory function in plants. For instance, H_2_S could promote seed germination of wheat and ameliorate copper-induced damage of plasma membrane integrity in root tips [11]. H_2_S has also been reported to counteract chlorophyll loss and reduce oxidative damage due to osmotic stress in sweet potato seedling leaves [12]. Furthermore, boron toxicity, chromium toxicity and cadmium toxicity in plants could be alleviated by H_2_S through enhancing the activities of antioxidant enzymes and decreasing the accumulation of toxic ions [13]–[16]. In addition, a low H_2_S concentration has been shown to promote the embryonic root length of *Pisum sativum* [17]. Similarly, our prior study has shown that H_2_S could enhance photosynthesis through promoting chloroplast biogenesis, photosynthetic enzyme expression and thiol redox modification in *Spinacia oleracea* seedlings [18]. Besides, our results have indicated that H_2_S plays an ameliorative role in protecting barley seedlings against aluminum toxicity by inducing the activities of antioxidant enzymes, increasing citrate secretion and the gene expression of citrate transporter, and enhancing the protein expression of PM H^+^-ATPase [19]. Interestingly, some evidences have recently demonstrated that H_2_S may delay senescence of cut flowers and prolong flower vase life in a wide spectrum of botanical species, including herbaceous and woody plants. In addition, H_2_S also could prolong the postharvest shelf life of strawberries and play an antioxidative role in fruits [20], [21]. However, at high concentrations, H_2_S may interfere with plant's normal growth and metabolism. For instance, high concentrations of H_2_S may impair photosynthetic electron transport and depress plant growth [22], [23].

Previous studies on H_2_S mainly focused on morphological, physiological and biochemical processes in plants. However, the detailed molecular mechanisms underlying plant response to H_2_S signal remain largely unknown. Moreover, none of the studies mentioned above have provided information on the changes of protein expression induced by physiological levels of H_2_S. Recently, proteomic approaches have emerged as a powerful tool for gaining insight into physiological changes at the cellular and biochemical level, allowing the function and regulation of a specific signaling molecule to be explored in detail. For instance, by using proteomic approaches, Bai *et al.* [24], found that G-protein coupled signaling is an early event that works upstream of NO biogenesis. Similarly, Lum *et al.* [25], investigated the downstream signaling pathways of NO in mung bean using a proteomic approach, suggesting that exogenous sodium nitroprusside (SNP), a donor of NO, could affect the expression level of photosynthetic enzymes and glucose metabolism. Therefore, comparative proteomic studies have been successfully applied to systematically investigate protein expression changes in several plant species to elucidate the roles of specific signaling molecules.

In the present study, we used a quantitative proteomic approach to identify global protein expression changes of *S. oleracea* seedlings under NaHS treatment, an exogenous H_2_S donor. Using this powerful tool, we observed extensive changes of protein expression relating to energy production and photosynthesis, cell rescue, development and defense and so on. The present results would provide some new insights into H_2_S-mediated metabolic and physiological changes in plants and also would accelerate the study of H_2_S signaling function in plants

## Materials and Methods

### Plant materials and growth conditions

Seeds of *Spinacia oleracea* were first sterilized in 75% ethanol for 3 min, then in 10% sodium hypochlorite solution for an additional 10 min followed by washing with distilled water and germinated in a soil/vermiculite (1∶1) mixture. Two-week-old seedlings were transferred to 1/2 Hoagland's solution (pH 6.0) in a controlled growth chamber with a light/dark regime of 15/9 h, relative humidity of 80%, temperature of 21/27°C and a photosynthetically active radiation (PAR) of 190 µmol m^−2^ s^−1^. NaHS was purchased from Sigma and used as an exogenous H_2_S donor as described by Hosoki *et al.*, [26]. The seedlings were treated with 100 µM NaHS for 30 d and the solution were changed every 3 d. The *S. oleracea* leaves were collected and immediately frozen in liquid N_2_ and stored at −80°C for subsequent experiments. Each experiment was repeated at least three times.

### Leaf area calculation, dry weight and relative water content analysis

Twenty leaves with the same leaf position were collected from control and NaHS treated seedlings, respectively, and then flatted on clean coordinate paper. Leaves were photographed with a digital camera at the same image resolution. Leaf area was calculated with pixels as described previously by Xiao *et al.* [27], using the Adobe Photoshop 7.0 software (Adobe Systems Inc., San Jose, CA). Each treatment comprised three biological replicates. Thus, the average leaf area was calculated from three replicates.

Leaves were removed from the same leaf position and immediately weighed to obtain the leaf fresh weight (FW). Leaves were subsequently placed into vials filled with distilled water for 24 h, then blotted to remove excess water and re-weighed to determine the leaf turgid weight (TW). Leaves were dried to a constant weight at 65°C and re-weighed to obtain the leaf dry weight (DW). Leaf relative water content (RWC) was calculated as (FW–DW)/(TW–DW)×100 [28].

### Pigment analysis, gas exchange and stomatal aperture measurements

Chlorophyll content was measured according to Lichtenthaler [29] with some modifications. After extraction using 10 ml of 80% (v/v) aqueous acetone, the content of total chlorophyll was calculated from the absorbance of leaf chlorophyll extracts at 470, 646 and 663 nm.

The net photosynthetic rate (*P*_n_) was measured using a portable photosynthesis system (Li-6400, Li-Cor, Lincoln, NE, USA) on the third fully developed leaf of each seedling. Air temperature, light intensity, CO_2_ concentration and air relative humidity were maintained at 25°C, 800 µmol m^−2^ s^−1^, 380 µl l^−1^, and 90%, respectively. *P*_n_ was expressed on a leaf area basis.

Measurements of stomatal apertures were performed as described by Desikin *et al.*, [30]. Abaxial epidermal strips from similar rosette leaves were floated in 10 mM 2-(N-morpholino)ethanesulfonic acid (MES) buffer (pH 6.15) containing 50 mM KCl and 50 µM CaCl_2_ for 2 h under light conditions to open the stomata before the addition of NaHS. Next, 100 µM NaHS was added to the buffer solution and incubated for another 2 h to assay stomatal aperture. Finally, stomatal aperture was calculated as the ratio of width to length using Sigma Scan Pro 5 software.

### Measurement of amino acid content

Amino acid analysis was carried out by ion exchange chromatography as described by Oliveria *et al.*, [31]. For each treatment, samples were ground in 50 ml of a methanol, chloroform, and water mixture (60∶25∶15 v/v/v) for 1 min. The ground samples were centrifuged, and then the clear supernatant was decanted into 100 ml beaker and allowed to partially evaporate overnight in a hood to remove the methanol and chloroform. The samples were taken to dryness in a vacuum desiccator and the dried extracts were suspended in 5 ml of citrate buffer (pH 2.2). The measurement of amino acid content was performed by an amino acid analyzer (Model L-8800, Hitachi Co. Ltd., Tokyo, Japan) with a column packed with Hitachi custom ion-exchange resin, which temperature were controlled from 30 to 70°C. The lithium citrate buffer and ninhydrin flow rates were 0.35 and 0.30 ml/min, respectively.

### Protein extraction and 2-DE analysis

Total proteins were extracted by the phenol procedure [32]. Briefly, one to two grams of the fresh leaves were ground in liquid nitrogen and total soluble proteins were extracted at 4°C for 1 h in 2 ml of 20 mM Tris-HCl buffer (pH 7.5) containing 250 mM sucrose, 10 mM ethylene diamine tetraacetic acid (EDTA), 1 mM phenylmethyl-sulfonyl fluoride (PMSF), 1% (w/v) Triton X-100, 5% β-mercaptoethanol and 1% (w/v) polyethylene polyvinyl pyrrolidone (PVPP). The homogenates were subjected to centrifugation at 12,000 *g*, 4°C for 15 min, after which the supernatants were added to two volumes of Tris-saturated phenol (pH 8.0) and the mixture was further vortexed for 30 min. Proteins were precipitated by adding five volumes of ammonium sulfate-saturated methanol and incubated at −20°C for at least 4 h. After centrifugation as described above, the protein pellets were re-suspended and rinsed with ice-cold methanol, followed by washing with ice-cold acetone twice, and spun down at 15,000 *g*, 4°C for 10 min after each washing. The final washed pellets were air-dried and dissolved in lysis buffer containing 8 M urea, 2 M thiourea, 4% (w/v) 3-[(3-Cholamidopropyl)dimethylammonio]-1-propanesulfonate (CHAPS), 1% (w/v) DL-dithiothreitol (DTT) and 1% (v/v) IPG buffer (pH 4–7). Protein concentrations were determined by the Bradford assay [33]. Two-dimensional electrophoresis (2-DE) was carried out according to Bjellqvist *et al.*, [34]. Samples containing 1.2 mg protein were loaded onto an IPG strip holder fitted with dry IPG strips (length 18 cm, pH 4–7) and rehydrated for 16 h at room temperature. Isoelectric focusing was carried out with an Ettan IPGphor system (GE Healthcare Amersham Bioscience, Little Chalfont, U.K.) using the following voltage program: 300 V for 1 h, 600 V for 1 h, 1000 V for 1 h, a gradient to 8000 V for 2 h, and then maintaining a voltage of 8000 V for 64000 V·h. Focused strips were then equilibrated by soaking in an equilibration solution (6 M urea, 30% glycerol, 2% SDS, 50 mM Tris-HCl, pH 8.8, and 1% DTT) for 15 min, followed by the same equilibration solution but with 2.5% iodoacetamide instead of DTT for another 15 min. Separation of proteins in the second dimension was performed on SDS-12.5% polyacrylamide gels. Each separation was repeated three times to ensure the protein pattern reproducibility.

### Gel staining, imaging and data analysis

SDS-PAGE gels were stained with Coomassie Brilliant Blue (CBB) R–250 and then scanned at 600 dots per inch (dpi) resolution using a scanner (Uniscan M3600, China). Gels were analyzed using PDQuest software (Version 7.0, Bio-Rad). For each gel, a set of three images was generated, corresponding to the original 2-D scan, the filtered image and the Gaussian image. The Gaussian image, containing three-dimensional Gaussian spots, was used for the quantification analysis. After normalization and background subtraction, a matchset was created by comparing the control gels. The intensity of each spot was determined using the spot quantification tool and expressed using the ratio of pixel intensity of a single spot to the pixel intensity of all spots on the gels. The intensity of each spot was normalized by the local regression model of the software to compensate for gel-to-gel variation [35]. The expression of a protein in a spot was defined as the relative pixel volume of that spot. Meanwhile, protein spots that changed by more than 2-fold and passed the Student's *t* test (*P*<0.05) were selected and identified by MALDI-TOF MS. To compensate for subtle differences in sample loading or gel staining/destaining during individual repeat experiments, We normalized spot volumes based on total intensity of valid spots were calculated for each 2-DE gel and used for statistical calculations of protein abundance. For some missing values, we manually checked, and a consistent reproducibility between normalized spot volumes was found in the three replicates (Table S1). Each experiment was repeated three times.

### In-gel protein digestion, protein identification and protein classification

Protein spots showing significant changes in abundance during the treatments were excised manually from colloidal CBB stained 2-DE gels and protein digestion with trypsin was performed as follows. Briefly, gel slices were first destained with a 1∶1 (v/v) solution of methanol and 50 mM NH_4_HCO_3_ for at least three times until the color of CBB was removed, then washed several times with water and completely dried in a vacuum centrifuge. Depending on protein amount, 2–3 µl of 0.1 mg µl^−1^ modified trypsin (Promega, sequencing grade) in 25 mM NH_4_HCO_3_ was added to the dehydrated gel spots. After 30 min incubation, 7 µl of 25 mM NH_4_HCO_3_ was added to submerge the gel spots and left at 37°C overnight. After digestion, the gel slices were washed with 0.1% trifluoroacetic acid (TFA) in 50% v/v acetonitrile (ACN) three times to acquire the peptides. Matrix was prepared by dissolving α-cyano-4-hydroxycinnamic acid (CHCA) in 50% ACN and 0.1% TFA. Ten microliters of matrix solution was added into the dry peptides, and vortexed for 30 min. 0.5 µl of peptide was mixed with 0.5 µl of matrix solution. A total of 1 µl of reconstituted in-gel digest sample was spotted onto an Anchorchip target plate. The dried sample on the target plate was washed twice with 1 µl of 0.1% TFA, and left for 30 s before solvent removal. MALDI-TOF MS analysis (ReFlexTMIII, Bruker) was used to acquire the peptide mass fingerprint (PMF). A standard peptide mixture was spotted adjacent to all samples for external calibration, followed by internal mass correction using peptide ions generated by trypsin autoprotolysis (*m*/*z* 842.5 and *m*/*z* 2211.10). Spectra were analyzed using the flexAnalysis software (Version 3.2, Bruker-Daltonics). Then, the measured tryptic peptide masses were transferred through the MS BioTool program (Bruker-Daltonics) as inputs to search against the taxonomy of green plants in the NCBI (NCBInr, downloaded on September 9, 2011) database. The parameters of PMF were as follows: 100 ppm tolerance as the maximum mass error, MH^+^ monoisotopic mass values, allowance of oxidation (M) modification, allowance for one missed cleavage, and fixed modification of cysteine by carboxymethyl (carbamidomethylation, C). Potential matches were identified by considering the Mascot score, the putative functions and differential expression patterns on 2-DE gels. Several criteria were used to assign a positive match with a known protein. These were as follows: (i) Protein identifications were validated manually, ensuring that at least 4 peptides matched. (ii) The coverage of protein sequences by the matching peptides had to reach a minimum of 10%. (iii) The score that was obtained from the analysis with the Mascot software indicated the probability of a true positive identification and had to be at least 70. Positive matches were BLAST searched against the UniPort (http://www.uniprot.org) and/or NCBI protein (http://www.ncbi.nlm.nih.gov) databases for updated annotation and identification of homologous proteins. The identified proteins were searched with the UniPort and TAIR databases to find out if their functions were known, then they were further classified using Functional Catalogue software (http://mips.gsf.de/projects/funcat).

### Statistical analysis

Values in figures were expressed as means ± SE. The statistical significance of the data was analyzed using an univariate analysis of variance (*P*<0.05) (one-way ANOVA; SPSS for Windows, Version 13.0). For proteomic experiment, protein samples for 2-DE gel image analysis were extracted from three independent seedlings grown in three different pots in the same growth chamber. Thus, for Control and H_2_S treatment, three independent biological replicates were performed in 2-DE gel image analysis. The ratio of H_2_S and CK in the table 1 was the average of three replicates. Statistic analysis for 92 protein spots on 2-DE gels was performed using Student's t-test (*P*<0.05) provided by PDQuest software as mentioned earlier.

**Table 1:** Identification of differentially expressed proteins in *Spinacia oleracea* after treatment with 100 µM NaHS.

Spot^a^	NCBI	Protein identity^c^	Thero.	Exper.	SC^f^	MP/TP^g^	M	C^h^	Quantitative changes	
	accession^b^		kDa/pI^d^	kDa/pI^e^			score		H_2_S/CK^i^	Species
**Amino acid, nitrogen and sulfur metabolism**										
42	gi|108862760	Glutathione synthetase, chloroplast precursor, putative, expressed	55/5.75	25/5.38	27%	8/28	72	U	1.51±0.20	*Oryza sativa Japonica Group*
58	gi|30683408	Class I glutamine amidotransferase domain-containing protein	40/5.3	20/5.39	31%	9/19	91	U	1.54±0.19	*Arabidopsis thaliana*
75	gi|195651721	Cysteine sulfinate desulfinase/cysteine desulfurase and related enzymes	31/8.44	25/5.95	43%	9/31	92	D	0.30±0.03	*Zea mays*
**C-compound and carbohydrate metabolism**										
13	gi|219810303	Cellulose synthase CesA10	30/4.82	24/5.22	27%	7/15	82	U	2.70±0.61	*Bambusa oldhamii*
27	gi|207059706	Caffeoyl CoA O-methyltransferase	28/4.88	29/6.11	30%	7/21	106	U	1.84±0.52	*Carthamus tinctorius*
57	gi|12322095	Trehalose-phosphatase, putative	41/9.04	39/6.84	21%	7/14	79	U	4.91±1.48	*Arabidopsis thaliana*
64	gi|145408196	Secondary wall-associated glycosyltransferase family 8D	61/8.92	28/5.51	25%	10/23	91	U	8.33±1.97	*Populus tremula x Populus alba*
73	gi|170102	Carbonic anhydrase precursor	28/5.74	28/5.91	47%	11/21	121	D	0.38±0.09	*Spinacia oleracea*
87	gi|302811518	Quasimodo1-like protein	58/9.24	19/5.45	30%	14/22	130	D	0.15±0.05	*Selaginella moellendorffii*
**Phosphate metabolism**										
4	gi|25137409	S-locus receptor kinase	50/8.17	16/4.8	13%	7/12	74	U	2.66±0.57	*Brassica oleracea*
32	gi|81075765	Ser/Thr protein kinase-like	47/8.82	16/5.76	21%	9/23	77	U	3.64±0.91	*Solanum tuberosum*
79	gi|179399401	Putative calcium dependent protein kinase	64/9.19	41/4.45	23%	11/25	99	D	0.04±0.01	*Silene diclinis*
**Energy production and photosynthesis**										
1	gi|170129	Rubisco activase precursor	52/6.28	45/5.53	36%	14/16	183	U	1.75±0.72	*Spinacia oleracea*
3	gi|306481796	Ribulose-1,5-bisphosphate carboxylase/oxygenase large subunit	49/6.34	17/4.69	14%	6/7	83	U	11.14±4.07	*Clematis sp. SH-2010*
7	gi|54303888	Ribulose-1,5-bisphosphate carboxylase/oxygenase large subunit	45/6.33	28/4.79	23%	7/7	114	U	13.37±3.39	*Panicum virgatum*
8	gi|49182654	Ribulose-1,5-bisphosphate carboxylase/oxygenase large subunit	15/6.43	33/4.81	25%	9/10	134	U	2.58±0.49	*Odontoschisma denudatum*
11	gi|15235029	Chlorophyll a-b binding protein CP26	30/6	26/5.18	35%	9/11	142	U	20.44±7.35	*Arabidopsis thaliana*
23	gi|255549948	Photosystem I reaction center subunit VI, chloroplast precursor, putative	15/9.99	24/5.49	50%	5/13	76	U	13.11±4.91	*Ricinus communis*
34	gi|170129	Rubisco activase precursor	52/6.28	40/5.7	16%	9/17	81	U	2.42±0.45	*Spinacia oleracea*
36	gi|255551591	NADH dehydrogenase, putative	12/7.56	18/5.83	66%	8/22	107	U	101.6±45.1	*Ricinus communis*
46	gi|131392	Oxygen-evolving enhancer protein 2, chloroplastic;	29/8.58	27/6.45	49%	11/22	142	U	3.23±0.43	*Spinacia oleracea*
47	gi|755801	ATP synthase	37/5.8	38/6.4	37%	11/33	127	U	2.57±0.09	*Spinacia oleracea*
48	gi|297842481	Thylakoid lumenal 29.8 kDa protein	28/6.17	41/6.45	28%	8/28	77	U	2.57±0.33	*Arabidopsis lyrata subsp. lyrata*
53	gi|298570223	Ribulose-1,5-bisphosphate carboxylase/oxygenase large subunit	52/6.23	27/6.79	26%	9/12	117	U	14.04±1.84	*Ochradenus baccatus*
54	gi|2392029	Chain L, activated spinach rubisco in complex with the product 3- phosphoglycerate	53/6.12	25/6.79	23%	10/13	129	U	15.68±4.11	*Spinacia oleracea*
55	gi|307548298	Phosphoenolpyruvate carboxylase	87/6.27	25/6.88	15%	9/19	74	U	17.79±4.58	*Panicum miliaceum*
61	gi|131392	RecName: Full = Oxygen-evolving enhancer protein 2, chloroplastic;	29/8.58	26/5.9	37%	8/22	111	U	1.22±0.09	*Spinacia oleracea*
**Lipid, fatty acid and isoprenoid metablism**										
24	gi|226496803	Serine palmitoyltransferase 2	54/9.04	24/5.34	14%	8/19	86	U	5.21±0.96	*Zea mays*
33	gi|194067759	Adenylate isopentenyltransferase	36/5.67	32/5.78	28%	6/11	77	U	21.01±9.83	*Ipomoea nil*
65	gi|255594379	Acyl-CoA dehydrogenase, putative	45/7.28	24/6.02	16%	6/9	74	U	5.76±0.62	*Ricinus communis*
68	gi|209402461	Putative plastid 1-deoxy-D-xylulose 5-phosphate reductoisomerase precursor	48/5.04	44/6.86	34%	8/23	79	D	0.29±0.04	*Mantoniella squamata*
69	gi|297847516	Lipase class 3 family protein	61/6.52	30/6.89	23%	9/19	92	D	0.11±0.03	*Arabidopsis lyrata subsp. lyrata*
84	gi|30687094	Cyclopropane-fatty-acyl-phospholipid synthase	99/6.05	41/5.22	8%	9/13	76	D	0.15±0.01	*Arabidopsis thaliana*
**Transcription, protein synthesis, folding, modification, destination**										
12	gi|30692594	Putative F-box/LRR-repeat protein 9	28/7.98	22/4.99	36%	6/14	78	U	1.99±0.62	*Arabidopsis thaliana*
17	gi|334183835	Small subunit ribosomal protein S1	57/5.06	12/5.41	27%	10/23	97	U	17.34±7.63	*Arabidopsis thaliana*
18	gi|170131	Ribosomal protein 30S subunit	34/6.69	35/6.37	40%	10/24	100	U	1.97±0.34	*Spinacia oleracea*
35	gi|255961421	Ribosomal protein L22	18/10.8	73/5.59	38%	6/14	99	U	13.83±2.22	*Dendrocalamus latiflorus*
40	gi|159470805	Peptidyl-prolyl cis-trans isomerase, FKBP-type	29/9.15	42/6.12	34%	9/35	82	U	3.06±0.55	*Chlamydomonas reinhardtii*
44	gi|255582427	Threonyl-tRNA synthetase, putative	76/7.63	16/6.39	24%	14/27	117	U	2.09±0.07	*Ricinus communis*
50	gi|77556384	F-box domain containing protein	59/6.58	51/6.15	22%	9/21	84	U	2.59±0.45	*Oryza sativa Japonica Group*
63	gi|302379151	PRP-like protein	17/5.12	27/4.78	47%	5/15	72	U	1.33±0.14	*Daucus carota*
70	gi|255539022	Skp1, putative	18/4.62	36/6.62	36%	7/23	81	D	0.01±0.003	*Ricinus communis*
76	gi|14150732	Hypersensitive-induced response protein	32/5.22	17/4.77	44%	8/19	92	D	0.22±0.01	*Oryza sativa*
88	gi|55296320	Putative DNA-(apurinic or apyrimidinic site) lyase	35/8.18	44/5.61	37%	9/31	91	D	0.27±0.06	*Oryza sativa Japonica Group*
92	gi|15222035	Two-component response regulator ARR15	23/5.83	28/5.72	43%	9/36	87	D	0.46±0.06	*Arabidopsis thaliana*
**Cell rescue, development and defense**										
2	gi|238814300	Pollen coat-like protein	4.64/5.96	15/4.59	100%	5/9	74	U	14.55±4.45	*Camellia sinensis*
6	gi|15240974	Glutaredoxin family protein	46/5.62	29/4.6	21%	7/14	75	U	2.87±0.57	*Arabidopsis thaliana*
10	gi|39841264	Phl p 3 allergen	11/8.94	15.4/4.99	67%	5/18	101	U	4.86±1.46	*Phleum pratense*
14	gi|626032	Lipoxygenase	103/6.06	28/5.27	12%	9/14	78	U	4.46±1.55	*Oryza sativa*
16	gi|302793903	Allene oxide synthase	52/6.35	14/5.38	16%	9/16	103	U	49.38±9.42	*Selaginella moellendorffii*
19	gi|224113557	cc-nbs-lrr resistance protein	135/6.19	24/5.43	16%	15/27	99	U	2.17±0.58	*Populus trichocarpa*
22	gi|1680686	Rust resistance kinase Lr10	72/6.34	33/5.56	22%	11/34	72	U	1.20±0.14	*Triticum aestivum*
26	gi|304325281	Rp1-like protein	139/6.35	34/5.52	11%	10/19	76	U	16.31±3.53	*Zea mays subsp. parviglumis*
28	gi|168068013	GLP5 GID1-like protein	47/6.14	43/5.44	15%	7/16	72	U	2.10±0.14	*Physcomitrella patens subsp. patens*
29	gi|50252814	Ethylene-forming enzyme-like	62/8.76	49/5.29	17%	7/31	73	U	3.64±0.87	*Oryza sativa Japonica Group*
30	gi|149939807	RPM1-interacting protein 4	24/9.24	21/5.63	29%	7/7	121	U	26.61±10.65	*Arabidopsis thaliana*
45	gi|15081223	Glycine-rich protein GRP17	53/10.4	26/6.28	29%	8/26	75	U	2.53±0.39	*Arabidopsis thaliana*
59	gi|156141675	Putative NBS domain resistance protein	19/7.08	16/5.49	29%	8/24	74	U	2.19±0.44	*Coffea spp. mixed genomic library*
80	gi|15808946	Auxin-regulated protein	50/6.64	12/5.81	21%	8/14	85	D	0.07±0.02	*Solanum lycopersicum*
**Cellular transport, transport facilities, transport routes and cellular signal transduction**										
5	gi|112145418	WRKY transcription factor 23	39/9.31	22/4.66	26%	7/11	93	U	2.71±0.75	*Hordeum vulgare subsp. vulgare*
31	gi|5834502	Potassium channel	95/6.82	21/5.71	9%	10/16	75	U	7.39±0.41	*Nicotiana paniculata*
37	gi|63094976	Phytochrome C	42/6.62	17/5.93	22%	9/17	80	U	4.62±0.48	*Pereskiopsis aquosa*
39	gi|18409228	Ninja-family protein AFP1	38/8.65	39/5.82	43%	10/29	88	U	10.42±2.48	*Arabidopsis thaliana*
43	gi|302771345	ABC transporter	76/9.74	58/5.79	29%	11/21	111	U	5.40±0.45	*Selaginella moellendorffii*
56	gi|18391384	SNARE-interacting protein KEULE	75/7.98	34/6.6	23%	13/33	83	U	9.24±2.56	*Arabidopsis thaliana*
90	gi|255080042	Mitochondrial carrier family	35/9.67	48/5.55	38%	11/35	113	D	0.43±0.06	*Micromonas sp. RCC299*
91	gi|308810769	K^+^-channel ERG and related proteins, contain PAS/PAC sensor domain (ISS)	77/6.47	18/5.61	24%	15/31	123	D	0.31±0.09	*Ostreococcus tauri*
**Protein with binding function or cofactor requirement and cellular components**										
15	gi|460989	beta tubulin	43/4.78	59/5.01	32%	11/13	172	U	13.24±5.41	*Oryza sativa Japonica Group*
20	gi|108864224	Endonuclease III-like protein 1, putative	40/9.64	17/5.48	35%	11/19	96	U	4.48±0.99	*Oryza sativa Japonica Group*
38	gi|226531021	Lipid binding protein	12/9.2	21/5.91	60%	6/16	73	U	5.73±0.18	*Zea mays*
41	gi|255553540	Protein binding protein, putative	83/8.85	20/5.13	15%	11/27	94	U	2.08±0.34	*Ricinus communis*
49	gi|18401203	Protein pleiotropic regulator PRL2	54/9.34	42/6.22	27%	9/18	95	U	5.93±0.37	*Arabidopsis thaliana*
51	gi|255541734	Structural maintenance of chromosome 1 protein, putative	85/5.6	19/6.81	16%	14/23	104	U	2.79±0.51	*Ricinus communis*
60	gi|164652942	14-3-3e protein	30/4.76	30/4.76	36%	7/8	123	U	1.77±0.52	*Gossypium hirsutum*
62	gi|126508572	14-3-3 protein Lil 1433-3	30/4.94	35/4.79	27%	8/14	104	U	1.48±0.36	*Lilium longiflorum*
78	gi|148878501	RecName: Full = Ribosome-inactivating protein PD-L3/PD-L4;	29/8.54	31/4.66	40%	7/18	118	D	0.50±0.08	*Phytolacca dioica*
85	gi|11094250	Cytosolic phosphoglucose isomerase	6.19/6.00	12/5.48	29%	11/27	99	D	0.38±0.09	*Arabidopsis thaliana*
89	gi|58013197	Actin	42/5.31	46/5.49	41%	10/29	94	D	0.42±0.05	*Isatis tinctoria*
**Function unknown and hypothetical proteins**										
9	gi|224092117	Predicted protein	39/9.62	21/5.21	23%	8/17	89	U	2.77±0.43	*Populus trichocarpa*
21	gi|49388823	Hypothetical protein	16/11.5	26/5.38	49%	6/11	81	U	5.16±1.21	*Oryza sativa Japonica Group*
25	gi|168005449	Predicted protein	44/4.57	26/6.21	27%	10/23	96	U	1.58±0.22	*Physcomitrella patens subsp. patens*
52	gi|18409257	Uncharacterized protein	43/5.71	17/6.75	19%	8/15	86	U	4.49±1.15	*Arabidopsis thaliana*
66	gi|168040725	Predicted protein	12/9.66	40/6.96	57%	6/19	82	D	0.42±0.06	*Physcomitrella patens subsp. patens*
67	gi|115461348	Os04g0678700	41/9.62	15/7	33%	11/22	111	D	0.23±0.01	*Oryza sativa Japonica Group*
71	gi|2058273	YK426	21/9.62	28/6.49	58%	9/29	91	D	0.38±0.02	*Oryza sativa (japonica cultivar-group)*
72	gi|293333271	Hypothetical protein LOC100383295	39/8.15	37/6.09	34%	10/29	96	D	0.43±0.05	*Zea mays*
74	gi|15239608	Uncharacterized protein	40/8.4	14/4.26	25%	8/17	87	D	0.19±0.04	*Arabidopsis thaliana*
77	gi|224082162	Predicted protein	17/9.47	20/4.78	52%	7/17	84	D	0.40±0.14	*Populus trichocarpa*
81	gi|224094680	Predicted protein	68/5.97	19/4.93	19%	10/17	90	D	0.12±0.02	*Populus trichocarpa*
82	gi|297832366	Hypothetical protein ARALYDRAFT_343373	121/7.96	24/5.15	17%	20/36	114	D	0.04±0.001	*Arabidopsis lyrata subsp. lyrata*
83	gi|116790018	Unknown	41/9.19	34/5.09	31%	10/17	115	D	0.34±0.07	*Picea sitchensis*
86	gi|115456089	Os03g0807800	30/10.2	18/5.31	46%	11/23	128	D	0.07±0.02	*Oryza sativa Japonica Group*

a Spot No. is the unique differentially expressed protein spot number which refers to the labels in Figure 3.

b Database accession numbers according to NCBInr.

c The name and functional categories of the proteins identified by MALDI TOF MS.

d Theoretical mass (kDa) and pI of identified proteins.

e Experimental mass (kDa) and pI of identified proteins.

f The amino acid sequence coverage for the identified proteins.

g Number of matched peptides (MP)/total searched peptides (TP).

h Up-regulated protein spots (U) or down-regulated protein spots (D).

i The quantitative changes ratio of H_2_S treatment and control.

Data are presented as the mean ± SE of three replicates.

## Results

### Effects of H_2_S on growth and photosynthesis of S. oleracea

**Figure 1:**
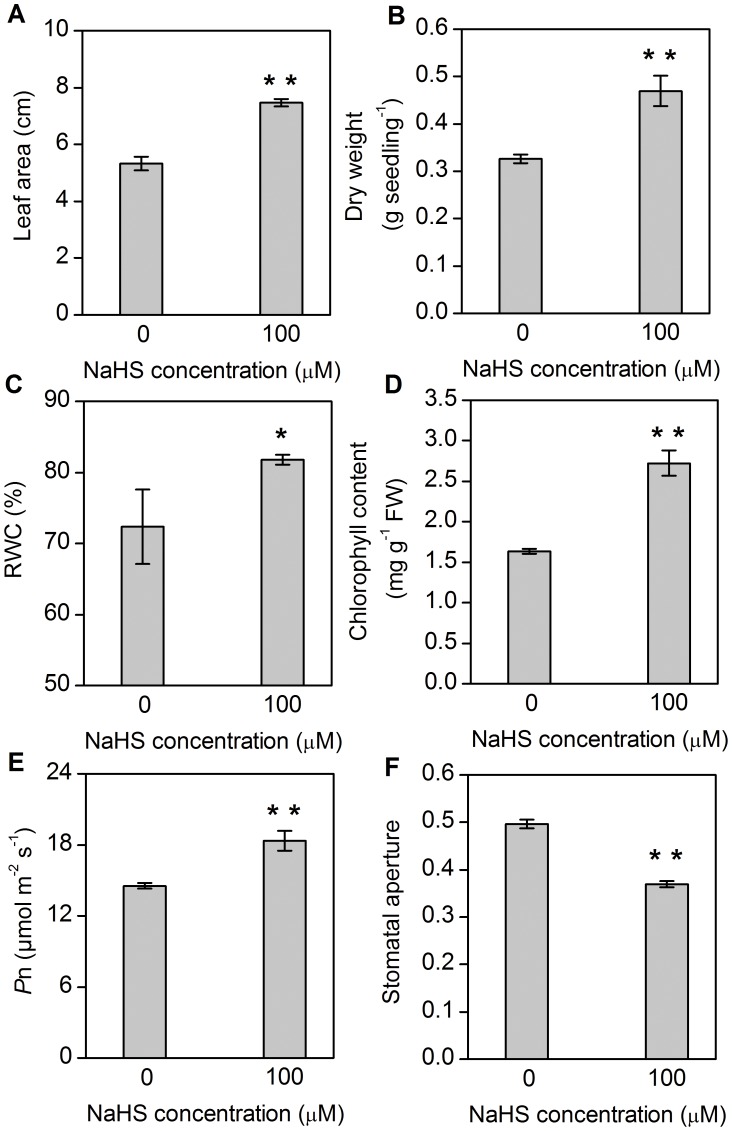
Effect of NaHS on leaf area (A), dry weight (B), relative water content (RWC) (C), chlorophyll content (D), photosynthesis (*P*_n_) (E) and stomatal aperture (F) in *Spinacia oleracea* leaves. Values of leaf area, dry weight, RWC and stomatal aperture are mean ± SE (*n* = 30), whereas values of *P*_n_ and chlorophyll content are mean ± SE (*n* = 4). The significant level of difference between control and treatment is indicated by * for *P*<0.05 and ** for *P*<0.01.

H_2_S could significantly affect growth and photosynthesis in *S. oleracea* seedlings. As shown in Fig. 1A, leaf area in *S. oleracea* seedlings treated with NaHS was profoundly increased (*P*<0.01) by 40% compared to control plants. Similarly, dry weight of *S. oleracea* seedlings under NaHS treatment was significantly increased (*P*<0.01) from 0.32 g per seedling to 0.47 g per seedling (Fig. 1B). The relative water content (RWC) of seedlings treated with NaHS was also increased (*P*<0.05) (Fig. 1C). In addition, chlorophyll content showed a remarkable increase (*P*<0.01) in seedlings treated with NaHS (Fig. 1D). Consistently, NaHS treatment obviously enhanced the photosynthetic rate of *S. oleracea* seedlings (*P*<0.01) (Fig. 1E). However, the stomatal aperture was reduced by around 20% in *S. oleracea* seedlings treated with NaHS (Fig. 1F).

### Changes in amino acid content in S. oleracea under NaHS treatment

**Figure 2:**
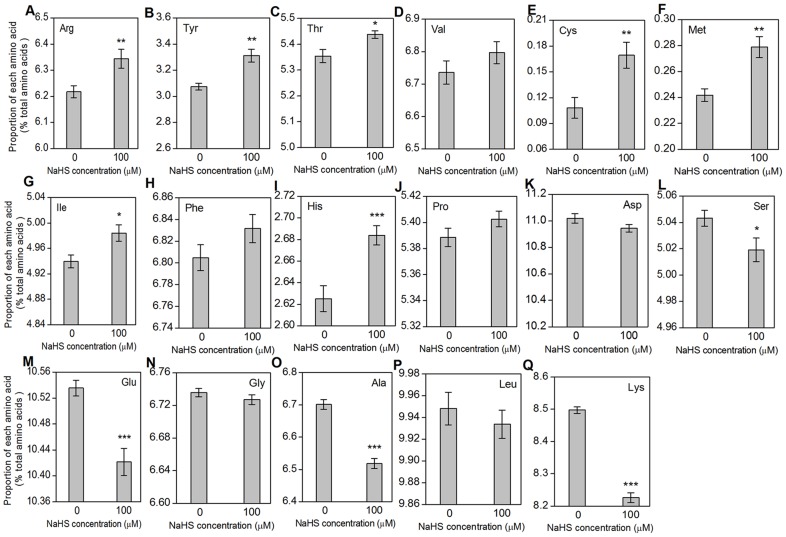
The amino acid content of *Spinacia oleracea* leaves treated with NaHS for 30 d. The values of amino acid content are mean ± SE (*n* = 3). The significant level of difference between control and treatment is indicated by * for *P*<0.05, ** for *P*<0.01 and *** for *P*<0.001.

To investigate whether a low concentration of H_2_S affected amino acid metabolism, we measured the contents of 17 kinds of amino acid in *S. oleracea* seedlings after NaHS treatment. As shown in Fig. 2, the contents of 10 amino acids, including Arg, Tyr, Thr, Val, Cys, Met, Ile, Phe, His and Pro, increased to some extent, whereas the contents of other seven amino acids, including Asp, Ser, Glu, Gly, Ala, Leu and Lys, decreased in *S. oleracea* seedlings under NaHS treatment.

### Differentially expressed proteins in S. oleracea under NaHS treatment

**Figure 3:**
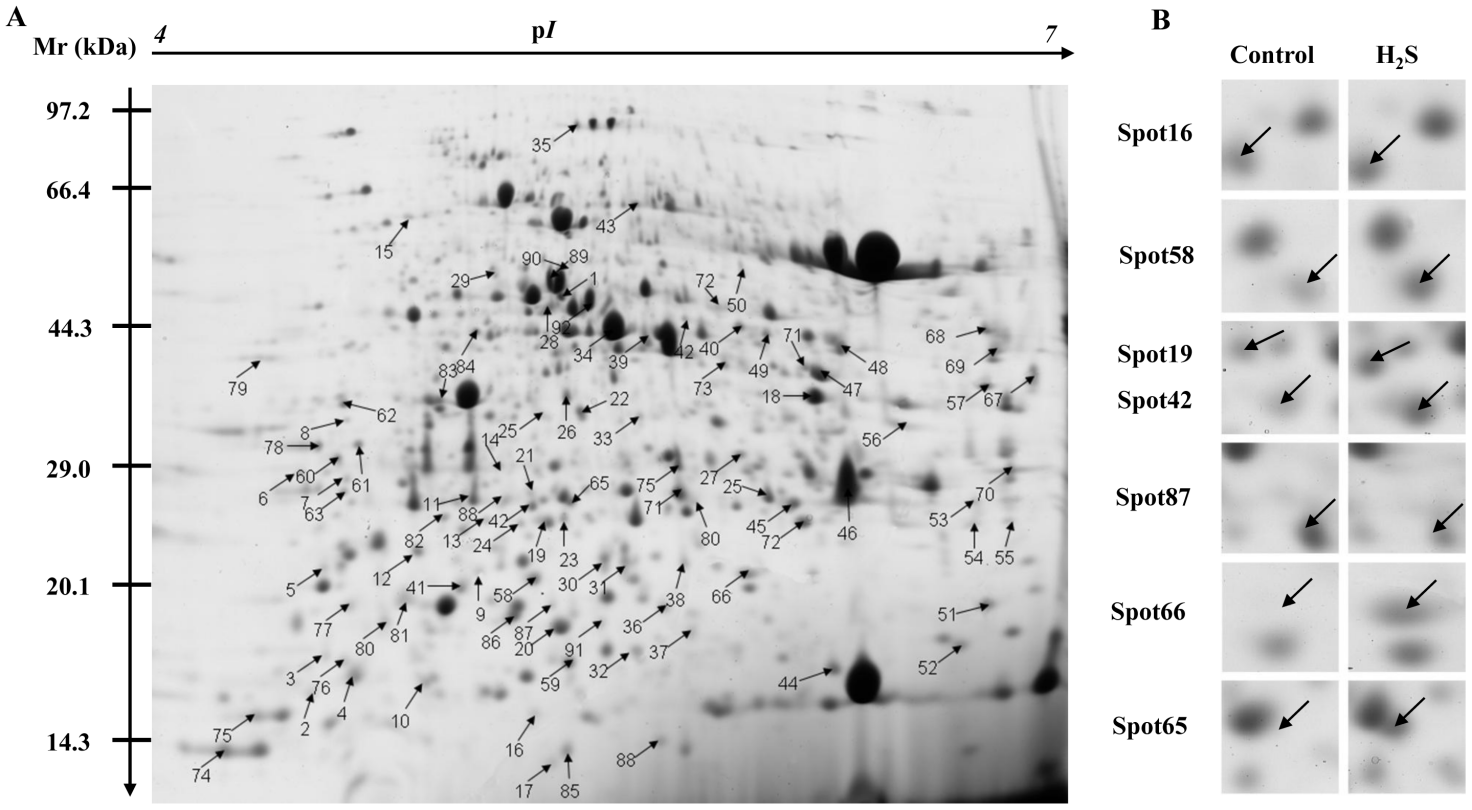
2D gel analysis of proteins extracted from *Spinacia oleracea* leaves. Molecular weight (MW) in kilodaltons and *p*I of proteins are indicated on the left and top of the gel, respectively. (A) Representative 2-DE gels of *Spinacia oleracea* in which 92 protein spots showing at least 2-fold changes (*P*<0.05) under NaHS treatment were identified by MALDI-TOF MS. (B) Close-up view of some differentially expressed protein spots.

To elucidate the possible mechanisms underlying H_2_S-induced increased in plant growth, we performed 2-DE to identify differences in the whole protein profiles of *S. oleracea* seedlings under 100 µM NaHS treatment compared with control plants. Representative images are presented in Fig. 3. The proteome was evaluated over an isoelectric point (*p*I) ranging from 4 to 7 and molecular weight (MW) ranging from 12 to 110 KDa (Fig. 3A). More than 1000 proteins were reproducibly resolved from the 2-D gels. Inspection of the gel patterns revealed that the MW and/or *p*I values of the spots differed from the theoretical values. Alternatively, some proteins were present in multiple spots, possibly due to translation from alternatively spliced mRNAs. Close-up views of several protein spots are shown in Figure 3B. A total of 92 proteins were positively identified by MALDI-TOF MS and listed in Table 1. Besides, the detail peptide information of identified proteins was listed in Table S2. Among these proteins, 65 protein spots were up-regulated and 27 were down-regulated in *S. oleracea* seedlings after H_2_S treatment.

### Reproducibility and variation in proteomics data

It was important to test the variation between biological replicates for both control and NaHS treatment sets. We carried out three biological replicates for our experiments (Table S1). Meanwhile, we also calculated for each protein the Pearson's linear correlation for protein abundant values across three biological replicates (Table 2). The reproducibility between the each two biological replicates of accumulation profiles of proteins showed a very high correlation (0.921 for CK-R1 and R2, 0.916 for CK-R2 and R3, 0.945 for CK-R1 and R3, 0.948 for H_2_S-R1 and R2, 0.931 for H_2_S-R2 and R3, 0.941 for H_2_S-R1 and R3). The reproducibility decreased to still significant levels with decreasing protein abundance (Table 2). In this study, we found that over 1000 protein spots were reproducibly resolved, of which the abundance of 92 spots was changed by at least 2-fold (sixty-five were up-regulated, whereas 27 were down-regulated) (Table 1). Besides, to further check the reproducibility of the replicates, we also analyzed the correlation of 92 differentially expressed proteins among three biological replicates (Figure S1), suggesting the well correlation and reproducibility among different biological replicates.

**Table 2:** Pearson's linear correlation for protein expression abundance values of *Spinacia oleracea* across three replicates.

	Control						H_2_S treatment					
Sum	R1-R2		R2-R3		R1-R3		R1-R2		R2-R3		R1-R3	
	n	Correlation	n	Correlation	n	Correlation	n	Correlation	n	Correlation	n	Correlation
		Coefficient		Coefficient		Coefficient		Coefficient		Coefficient		Coefficient
>1000	55	0.921	56	0.916	57	0.945	68	0.948	69	0.931	66	0.941
500 to 1000	88	0.746	80	0.815	84	0.798	93	0.845	98	0.832	95	0.851
100 to 500	231	0.639	261	0.713	278	0.721	287	0.726	298	0.745	294	0.765
50 to 100	258	0.631	247	0.659	297	0.661	256	0.766	274	0.726	264	0.729
20 to 50	307	0.678	298	0.645	312	0.648	301	0.691	287	0.687	298	0.698
10 to 20	149	0.621	151	0.603	144	0.615	145	0.615	165	0.625	155	0.639
5 to 10	54	0.602	59	0.615	59	0.598	58	0.588	50	0.601	51	0.612
<5	38	0.584	31	0.498	38	0.554	37	0.521	32	0.514	29	0.509

Sum stands for protein abundance and n stands for the number of protein in this abundant interval.

### Functional classification of differentially expressed proteins

**Figure 4:**
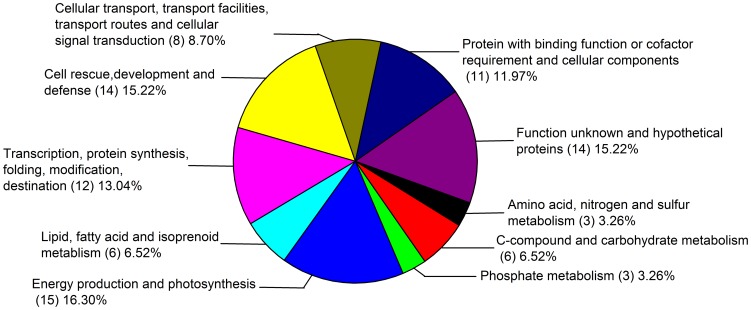
Outline of biological functional classification of the all identified proteins (92) including the up-regulated and down-regulated by H_2_S. Each identified protein listed in Table 1 was functionally classified according to their known and putative functions. The proportion of identities in each functional category was the sum of the proportion of all identities.

Among the 92 identified proteins, 14 proteins had unknown functions or were hypothetical proteins. However, 78 had assigned functions and could be classified into 9 groups based on their biochemical function (Table 1, Fig. 4). The majority of the protein profile corresponded to energy production and photosynthesis associated proteins (16.30%), followed by cell rescue, development and defense related proteins (15.22%). In addition, transcription, protein synthesis, folding and modification related proteins (13.04%) and proteins with binding functions, cofactor requirements and cellular components (11.97%) took a large part of the identified proteins. Cellular signal transduction (8.70%) and metabolism related proteins (amino acid, nitrogen and sulfur-related protein were 3.26%, C-compound related protein was 6.52% and phosphate-related protein was 3.26%) were also found in our present study (Fig. 4, Table 1).

## Discussion

H_2_S has recently been discovered to be an important signaling molecule involved in many different plant physiological processes, including seed germination [11], root organogenesis [17], abiotic stress tolerance [13], [14], [36], [37], photosynthesis [18] and guard cell movement [38], [39], senescence of cut flowers [20] and postharvest shelf life of fruits [21]. Although the functional roles of H_2_S have been studied in these physiological processes, the exact mechanisms by which H_2_S transmits the signal are still unclear. Therefore, our present study aimed to investigate H_2_S responsive differentially expressed proteins in plants using a proteomic approach.

### H_2_S affected energy production and photosynthesis-associated protein expression

Carbon dioxide fixation is an essential process of photosynthesis, and this pathway involves in many enzymes that catalyze and regulate energy generation [40]. In this study, we identified several photosynthesis-associated proteins and their expression levels were significantly up-regulated after NaHS treatment, e.g., the ribulose-1,5-bisphosphate carboxylase/oxygenase large subunit (rubisco LSU, spots 3, 7, 8 and 53) and rubisco activase precursor (spots 1 and 34). Rubisco, the CO_2_ fixing enzyme in Calvin cycle, is the primary limiting factor of net photosynthesis. H_2_S could obviously increase the protein expression of rubisco LSU. Similarly, we have previously reported that the activity of rubisco and protein and gene expression of rubisco LSU were significantly enhanced after 100 µM NaHS treatment [18]. Here, proteomic results confirmed that the increased expression of rubisco LSU and Rubisco activase precursor by H_2_S may enhance Calvin cycle activity, promoting the increment of photosynthetic CO_2_ assimilation and plant growth. Besides, we also detected that phosphoenolpyruvate carboxylase (PEPc, spot 55), which plays a key role in photosynthetic CO_2_ assimilation and plant growth, was up-regulated after H_2_S treatment. This result is consistent with protein expression data obtained using the western blotting method (data not shown). Similarly, these results were supported by physiological measurements (Fig. 1E).

It's well known that large amounts of ATP are needed by plants to provide sufficient energy for growth, development and photosynthesis [41], [42]. Therefore, ATP synthase and ATPase are key enzymes in energy production and conversion. In this study, we identified ATP synthase (spot 47) and found that its expression was up-regulated by H_2_S, suggesting that H_2_S treatment could increase energy production which could be utilized for the growth and development of plants. We also identified another important protein, chlorophyll a/b binding protein CP26 (LHCB, spot 11), whose expression was significantly increased under NaHS treatment. The LHCB proteins are the apoproteins of the light-harvesting complex of photosystem II (PSII), which are normally complexed with chlorophyll and xanthophylls and serve as the antenna complex [43]. Therefore, high expression of LHCB may increase PSII activity and electron transfer efficiency. Similarly, our previous results have shown that the maximal photochemical efficiency of PSII (*F*_v_/*F*_m_) is reached in the presence of H_2_S [18]. In other words, H_2_S appears to have a positive effect on the process of light capture in photosynthesis.

### H_2_S affected cell rescue, development and defense-related protein expression

The gaseous hormone ethylene plays multiple roles in regulating plant growth and development [44]. Ethylene is produced biologically from *S*-adenosylmethionine (SAM) via the following pathway: SAM→1-aminocyclopropane-1-carboxylic acid (ACC)→ethylene [44]. Ethylene-forming enzyme (EFE) is responsible for oxidation of ACC to ethylene [44], [45]. Here, we identified an ethylene-forming enzyme-like protein (spot 29) whose abundance was obviously increased after H_2_S treatment, indicating that ethylene biosynthesis may also be associated with H_2_S signaling pathway. Interestingly, Liu *et al.* [46], have reported that exogenous application of ethylene could significantly increase endogenous H_2_S content in *Arabidopsis* seedlings. Meanwhile, they also found that the generation of H_2_S induced by NO might mediate ethylene-induced stomatal closure in *Arabidopsis* [46]. Similarly, it's well known that jasmonic acid (JA) is involved in a wide range of stress, defense and development processes in plants [47]. In our study, we identified allene oxide synthase (AOS, spot 16), which is involved in the biosynthesis of JA. Moreover, AOS abundance significantly increased under H_2_S treatment, suggesting that H_2_S could enhance plant defense by increasing the level of JA. Interestingly, Hou *et al.* [48], have reported that H_2_S may function downstream of H_2_O_2_ in JA induced-stomatal closure of *Vicia faba*. In addition, JA could enhance the generation of endogenous H_2_S and L-cysteine desulfhydrase activity in guard cells of *Vicia faba* leaves. Therefore, we hypothesized that the signaling pathway of JA may also be associated with H_2_S signaling networks. Lipoxygenases (LOXs) are non-heme iron-containing dioxygenases widely distributed in plants and animals [49]. Moreover, LOXs may be involved in a number of diverse aspects of plant physiology including growth and development, pest resistance, and senescence or responses to wounding [49]. Besides, LOXs are required for the wound-induced biosynthesis of JA in leaves [49]. Here, we detected LOX (spot 14) and its protein expression level was obviously increased after H_2_S treatment. Therefore, we speculated that H_2_S signaling function may be related with JA pathway to some extent. Glutaredoxins (Grxs) are small oxidoreductases of the thioredoxin family of proteins regulating the thiol redox state of several proteins [50]. Thereby, Grxs play key roles in different aspects of plant development and defense through regulating and maintaining thiol redox homeostasis. In our study, we identified glutaredoxin family protein (spot 6) and found that H_2_S treatment obviously up-regulated this protein expression, indicating that H_2_S may be involved in thiol redox modification and cell redox homeostasis, in line with our previously published paper [18].

In this study, several proteins involved in plant response to biotic and abiotic stresses were identified under H_2_S treatment. For instance, cc-nbs-lrr resistance protein (spot 19), putative NBS domain resistance protein (spot 59), Rp1-like protein (spot 26) and RPM1-interacting protein 4 (spot 30). These proteins are essential regulators of plant defenses and play central roles in resistance against infection by pathogens [51]. Our results suggest that H_2_S could protect plants from pathogen infection by increasing these protein abundances. Further, glycine-rich proteins (GRPs), containing >60% glycine, have been found in the cell walls of many higher plants and form a group of structural protein components of the wall in addition to extensins and proline-rich proteins [52]. GRPs play very important roles in the post-transcriptional regulation of gene expression in plants under various stress conditions, in most cases, they are accumulated in the vascular tissues and their synthesis is a part of the plant's defense mechanism [53]. Interestingly, we also identified this important protein, glycine-rich protein GRP17 (spot 45), whose abundance obviously increased under H_2_S treatment.

Pollination and elongation of the pollen tube are important processes for the normal growth and development of flower plants [54]. Pollen coat protein (PCP) plays a multiplicity of roles in the pollination process [55]. In our study, we identified a pollen coat-like protein (spot 2) and Phl p 3 allergen (spot 10), both of which were up-regulated by H_2_S. Therefore, we hypothesized that H_2_S might be involved in the pollination process and elongation of the pollen tube and this physiological function of H_2_S is the same as that of NO signaling molecule in flower plants [54].

### H_2_S affected substance metabolism, lipid, fatty acid and isoprenoid metabolism related protein expression

Substance metabolism is the basic life activity and is vulnerable to environmental stresses in plants. As shown in Fig. 1A and 1B, leaf area and dry weight were obviously increased in *S. oleracea* seedlings treated with 100 µM NaHS, a donor of H_2_S. These results showed that H_2_S affected plant growth and the accumulation of organic compounds. In addition, it's well known that aminotransferase and methyltransferase are key links between carbon and nitrogen metabolism [56], [57]. Here, we found that glutathione synthetase (spot 42), class I glutamine aminotranferase (spot 58) and caffeoyl CoA O-methyltransferase (spot 27) were all up-regulated under NaHS treatment. Among them, class I glutamine aminotranferase is known to be involved in L-methionine (Met) biosynthesis, suggesting that high expression of this protein may increase the formation of Met, which was completely consistent with Met content (Fig. 2F). Besides, the up-regulated of caffeoyl CoA O-methyltransferase and down-regulated of cysteine desulfurase (spot 75) may increase the cysteine (Cys) content, as supported by our previously published data on Cys and GSH content [18].

Carbon compounds in the cell wall serve as an important physical barrier, having a significant role in cell defense against various external stresses [58]. Conventionally, accumulation of carbon compounds, such as callose and lignin, has been considered to be one of the most important defense mechanisms. In the present study, we identified cellulose synthase CesA10 (spot 13) and secondary wall-associated glycosyltransferase family 8D (spot 64) and found that their expressions significantly increased after NaHS treatment. Both CesA10 and secondary wall-associated glycosyltransferase family 8D are known to be important for cellulose synthesis and lignin biosynthesis [59]. Therefore, we speculated that H_2_S may play a crucial role in plant defense by increasing the biosynthesis of cell wall related compounds.

We also identified several lipid, fatty acid and isoprenoid metabolism related proteins, including three up-regulated proteins, i.e., serine palmitoyltransferase 2 (spot 24), adenylate isopentenyltransferase (spot 33) and putative acyl-CoA dehydrogenase (spot 65), and three down-regulated proteins, i.e., putative plastid 1-deoxy-D-xylulose 5-phosphate reductoisomerase precursor (spot 68), lipase class 3 family protein (spot 69) and cyclopropane-fatty-acyl-phospholipid synthase (spot 84). These results indicate that H_2_S may function as a signaling molecule in lipid, fatty acid and isoprenoid metabolic pathways. As we known, in plants, acyl-CoA dehydrogenase is involved in the β-oxidation process of fatty acid [60]. Our results suggested that H_2_S may be involved in regulating the β-oxidation process of fatty acid by changing the protein expression of acyl-CoA dehydrogenase.

### H_2_S was involved in transcription, protein synthesis, folding modification and destination related processes

Regulation of gene expression is achieved at several levels, i.e., transcriptional, post-transcriptional, translational, and post-translational. Several proteins implicated in transcription, protein synthesis and modification were identified in the present study, including three ribosomal proteins (spots 17, 18, 35), PRP-like protein (spot 63), putative Skp1 (spot 70), hypersensitive-induced response (HIR) protein (spot 76), two-component response regulator ARR 15 (spot 92). Among them, three ribosomal proteins, whose abundances were increased following H_2_S treatment, indicated that H_2_S signaling molecule could accelerate the biosynthesis process of protein in plants. PRP-like protein, which is involved in mRNA processing and regulation of the timing of the transition from a vegetative to reproductive phase in plants, was also found to be obviously increased under H_2_S treatment. Besides, Skp1 is a core component of the Skp1-Cullin-F-box (SCF) family of E3 ubiquitin ligases and serves to tether the rest of the complex to an F-box protein, which provides specificity for binding ubiquitin ligase substrate proteins and plays a role during embryogenesis and early postembryonic development, especially during cell elongation and division [61]. In our experiment, the protein expression of Skp1 was decreased following H_2_S treatment, indicating that H_2_S could inhibit protein degradation and delay embryogenesis in plant. In addition, HIR proteins are a group of proteins involved in hypersensitive reaction (HR). They belong to the PID (proliferation, ion and death) superfamily, whose members function in cell proliferation, ion channel regulation and cell death [62]. HIR protein expression in maize and barley is associated with localized host cell death and disease resistance responses [62], [63]. Here, we detected a HIR protein and found that expression of this protein was clearly decreased after H_2_S treatment, indicating that H_2_S may act as a signaling molecule that regulates the plant immunity by changing the expression of HIR protein. Finally, the two-component response regulator ARR 15 is transcriptional activator that binds specifically to the DNA sequence 5′-[AG]GATT-3′ and functions as a response regulator involved in the His-to-Asp phosphorelay signal transduction system for cytokinin and meristem stem cell maintenance [64]. We found that the protein expression of ARR 15 was obviously changed after H_2_S treatment, suggesting that H_2_S may be involved in His-to-Asp phosphorelay signal transduction system.

### H_2_S affected cellular transport, transport facilities, transport routes and cellular signal transduction related protein expression

It's well known that WRKY transcription factors (WRKY TFs) are a large family of regulatory proteins involved in various plant processes but most notably in coping with diverse biotic and abiotic stresses [65], [66]. In our study, we identified WRKY TF 23 (spot 5) and found that its abundance was obviously increased after H_2_S treatment, indicating that H_2_S-enhanced plant immunity may be associated with the regulation of WRKY TFs. Besides, a previous study in animal research has shown that H_2_S, as an endogenous gaseous signal molecule, could induce the opening of K_ATP_ channels [3]. However, it is not clear whether H_2_S could affect potassium channels in plants. Interestingly, in our experiment, we found that H_2_S increased the protein expression of potassium channels (spot 31) in *S. oleracea* seedlings. Moreover, our unpublished data also has shown that H_2_S could increase K content and promote K^+^ influx in barley seedlings roots under high salt stress by changing the expression of K channel related genes. In addition, García-Mata and Lamattina have reported that H_2_S could induce stomatal closure and participate in the ABA-dependent signaling pathway, possibly through the regulation of ABC transporter proteins in guard cells [38]. This result is surprisingly consistent with our proteomic data, because we also identified an ABC transporter protein (spot 43) and found that its expression was obviously increased under H_2_S treatment. It's well known that phytochromes are a family of photoreceptors that modulate the expression of a large number of light-responsive genes and control plant growth and development [67]. Recently, phytochrome has been found to regulate various biotic and abiotic stresses, such as salinity, drought, cold and herbivory [68]. Cross-talk between phytochrome-mediated light signals and some other signaling pathways has been reported in diverse plants [69]. Thus it is possible that phytochrome is involved in H_2_S signaling pathway. In our study, we identified phytochrome C (spot 37) and found that its expression was up-regulated under H_2_S treatment, suggesting that H_2_S could promote plant growth and development and these processes were associated with the signal pathway of phytochrome. Further research need to widen our understanding of the relationship between phytochrome and H_2_S signal in plants.

### H_2_S affected proteins with binding function or cofactor requirements and cellular components related protein expression

In the present study, we identified several proteins with binding function or cofactor requirement and cellular components, including beta tubulin (spot 15), lipid binding protein (spot 38), protein binding protein (spot 41), 14-3-3e protein (spot 60), 14-3-3 protein Lil 1433-3 (spot 62), cytosolic phosphoglucose isomerase (spot 85) and actin (spot 89). Among them, actin and tubulin dynamics have important functions in cellular homeostasis. H_2_S treatment affected actin and tubulin expressions, suggesting that H_2_S might be involved in cellular homeostasis. 14-3-3 proteins are known to bind certain phosphorylated proteins to complete phosphoregulation events. In plants, 14-3-3 protein binding activity includes the regulation of key metabolic enzymes, such as nitrate reductase and sucrose synthase [70], [71], and the activation of plasma membrane H^+^-ATPase [72]. In addition, 14-3-3 proteins also participate in a wide array of signal transduction regulatory events [73]. In our experiment, we identified two 14-3-3 proteins and found that their expression levels were obviously increased after H_2_S treatment, indicating that H_2_S may function in the signal transduction regulatory events involving 14-3-3 protein. However, the detailed mechanism of cross-talk between H_2_S and 14-3-3 protein in signal transduction regulatory events is still unclear.

**Figure 5:**
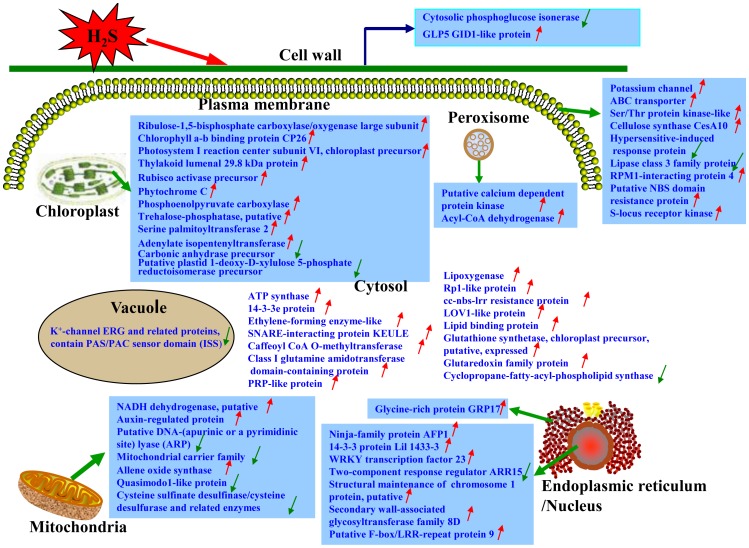
Schematic model of response mechanism in *Spinacia oleracea* leaves treated with NaHS. Some of the H_2_S-responsive proteins are indicated, with those up-regulated marked by red “
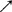
” and those down-regulated marked by green “
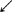
”.

In summary, by using a comparative proteomic strategy, we compiled an overview of the systematic mechanism by which *S. oleracea* seedlings respond to the H_2_S signaling molecule. Quantitative analysis of more than 1000 highly reproducible proteins on 2-DE profiles identified 92 proteins which expressions were significantly changed in response to H_2_S. These proteins were classified into 9 functional groups, the main one being energy production and photosynthesis associated proteins, followed by cell rescue, development and defense related proteins. In addition, transcription, protein synthesis, folding, modification related proteins and proteins with binding function or cofactor requirement and cellular components formed a large part of the identified proteins. Cellular signal transduction and metabolism related proteins were also found. Taken together, the above-mentioned results indicated that H_2_S played important roles in a set of proteins associated with energy production, photosynthesis, metabolism, cell rescue, cell defense and protein synthesis, folding and signal transduction, etc. On the basis of above results, we also studied another important question regarding H_2_S signaling in plant cells, the localization of its sub-cellular target, and proposed a schematic model of systematic response mechanism of *S. oleracea* seedlings to H_2_S (Fig. 5). We found that several sub-cellular organelles were predominantly affected by H_2_S or H_2_S signaling in plants, including mitochondria, chloroplast, nucleus, and peroxisome. This research provides valuable information about the response of plants to H_2_S as a signaling molecule.

## Supporting Information

Figure S1 **Results of the correlation analysis of three control replicates and three NaHS treatment replicates in** ***Spinacia oleracea*** **leaves.** Scatter plots of the 92 differentially expressed proteins quantitation Log_10_^(CK-R1)^ and Log_10_^(CK-R2)^ ratio (A), Log_10_^(CK-R2)^ and Log_10_^(CK-R3)^ ratio (B), Log_10_^(CK-R1)^ and Log_10_^(CK-R3)^ ratio (C), Log_10_^(H2S-R1)^ and Log_10_^(H2S -R2)^ ratio (D), Log_10_^(H2S –R2)^ and Log_10_^(H2S –R3)^ ratio (E), Log_10_^(H2S -R1)^ and Log_10_^(H2S –R3)^ ratio (F), with correlation coefficients of 0.984, 0.985, 0.981, 0.982, 0.979 and 0.977, respectively.
(DOC)

Table S1 **Spot volumes of differentially expressed protein (92) of** ***Spinacia oleracea*** **with NaHS treatment for 30 d.** Labeled R1, R2 and R3 stand for Replicate 1, Replicate 2 and Replicate 3 for Control or NaHS treatment, respectively.
(DOC)

Table S2 **Details of identified proteins (92) and peptides list of each protein in** ***Spinacia oleracea*** **leaves after treatment with 100 µM NaHS.**
(DOC)
