# Supplementary material for: Comparative Proteomic Analysis of Differentially Expressed Proteins Induced by Hydrogen Sulfide in Spinacia oleracea Leaves
Source: PLoS One. 2014 Sep 2;9(9):e105400. doi: 10.1371/journal.pone.0105400 (PMC4152154; doi:10.1371/journal.pone.0105400)

**Figure S1** Results of the correlation analysis of three control replicates and three NaHS treatment replicates in *Spinacia oleracea* leaves. Scatter plots of the 92 differentially expressed proteins quantitation Log_10_ ^(CK-R1)^ and Log_10_ ^(CK-R2)^ ratio (A), Log_10_ ^(CK-R2)^ and Log_10_ ^(CK-R3)^ ratio (B), Log_10_ ^(CK-R1)^ and Log_10_ ^(CK-R3)^ ratio (C), Log_10_ ^(H2S-R1)^ and Log_10_ ^(H2S -R2)^ ratio (D), Log_10_ ^(H2S –R2)^ and Log_10_ ^(H2S –R3)^ ratio (E), Log_10_ ^(H2S -R1)^ and Log_10_ ^(H2S –R3)^ ratio (F), with correlation coefficients of 0.984, 0.985, 0.981, 0.982, 0.979 and 0.977, respectively.


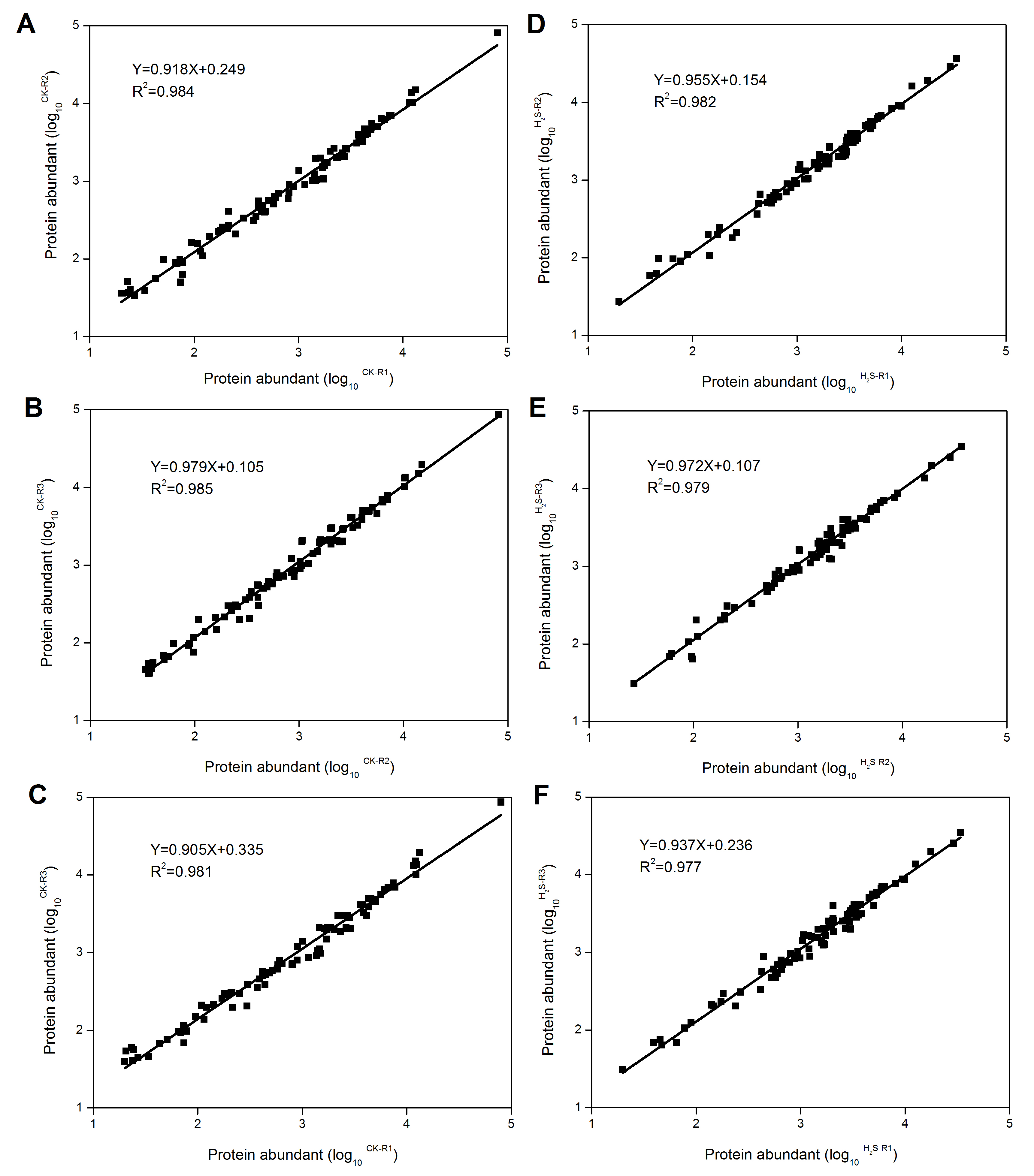

Supplement: Figure S1 — Results of the correlation analysis of three control replicates and three NaHS treatment replicates in Spinacia oleracea leaves. Scatter plots of the 92 differentially expressed proteins quantitation Log10 (CK-R1) and Log10 (CK-R2) ratio (A), Log10 (CK-R2) and Log10 (CK-R3) ratio (B), Log10 (CK-R1) and Log10 (CK-R3) ratio (C), Log10 (H2S-R1) and Log10 (H2S -R2) ratio (D), Log10 (H2S –R2) and Log10 (H2S –R3) ratio (E), Log10 (H2S -R1) and Log10 (H2S –R3) ratio (F), with correlation coefficients of 0.984, 0.985, 0.981, 0.982, 0.979 and 0.977, respectively. (DOC) [file pone.0105400.s001.doc]
