# Supplementary material for: Comparative Proteomic Analysis of Differentially Expressed Proteins Induced by Hydrogen Sulfide in Spinacia oleracea Leaves
Source: PLoS One. 2014 Sep 2;9(9):e105400. doi: 10.1371/journal.pone.0105400 (PMC4152154; doi:10.1371/journal.pone.0105400)
Supplement: Table S1 — Spot volumes of differentially expressed protein (92) of Spinacia oleracea with NaHS treatment for 30 d. Labeled R1, R2 and R3 stand for Replicate 1, Replicate 2 and Replicate 3 for Control or NaHS treatment, respectively. (DOC) [file pone.0105400.s002.doc]

**Table S1** Spot volumes of differentially expressed protein (92) of *Spinacia oleracea* with NaHS treatment for 30 d. Labeled R1, R2 and R3 stand for Replicate 1, Replicate 2 and Replicate 3 for Control or NaHS treatment, respectively.

|  | **Control** | | |  | **H_2_S treatment** | | |
| --- | --- | --- | --- | --- | --- | --- | --- |
| **spot** | **R1** | **R2** | **R3** |  | **R1** | **R2** | **R3** |
| 42 | 2012 | 2434.7 | 1986 |  | 3086.8 | 3125 | 3326 |
| 58 | 2628 | 2120 | 2087 |  | 3277 | 3520 | 3601 |
| 75 | 1878 | 1730 | 2103 |  | 594 | 560 | 534 |
| 13 | 472 | 401 | 556 |  | 1041 | 1354 | 1401 |
| 27 | 1457 | 1025 | 1109 |  | 1862 | 2019 | 2534 |
| 57 | 415 | 553 | 569 |  | 2714 | 2015 | 2594 |
| 64 | 213 | 410 | 305 |  | 2506 | 2019 | 2534 |
| 73 | 6184 | 6354 | 6487 |  | 2993 | 2301 | 2003 |
| 87 | 441 | 403 | 387 |  | 45 | 62 | 75 |
| 4 | 1411 | 1234 | 1054 |  | 3000 | 3211 | 3432 |
| 32 | 902 | 845 | 1203 |  | 3816 | 3451 | 3120 |
| 79 | 799 | 602 | 706 |  | 19.8 | 26.9 | 31 |
| 1 | 894 | 845 | 801 |  | 1655 | 1502 | 1302 |
| 3 | 26.8 | 34 | 45 |  | 413 | 364 | 329 |
| 7 | 33.7 | 39 | 46 |  | 571 | 506 | 469 |
| 8 | 78 | 63 | 97 |  | 173 | 198 | 231 |
| 11 | 179 | 231 | 298 |  | 5033 | 4564 | 4010 |
| 23 | 66 | 89 | 97 |  | 1229 | 1023 | 894 |
| 34 | 11564 | 10231 | 13215 |  | 29136 | 28749 | 25423 |
| 36 | 20.6 | 36 | 54 |  | 3150 | 3012 | 3684 |
| 46 | 12315 | 10234 | 10236 |  | 33860 | 36523 | 34652 |
| 47 | 3874 | 3605 | 3260 |  | 9920 | 8950 | 8701 |
| 48 | 2329 | 2019 | 1877 |  | 5329 | 5019 | 5498 |
| 53 | 115 | 126 | 139 |  | 1744 | 1894 | 1657 |
| 54 | 43 | 56 | 67 |  | 873 | 801 | 834 |
| 55 | 69 | 87 | 93 |  | 1584 | 1406 | 1325 |
| 61 | 7425 | 7041 | 7901 |  | 9411 | 9021 | 8795 |
| 24 | 208 | 245 | 306 |  | 1280 | 1039 | 1598 |
| 33 | 24.5 | 39.9 | 56 |  | 786 | 704 | 745 |
| 65 | 606 | 615 | 789 |  | 3398 | 3960 | 4120 |
| 68 | 5673 | 5021 | 5601 |  | 1580 | 1630 | 1409 |
| 69 | 2642 | 2312 | 2069 |  | 181 | 246 | 296 |
| 84 | 1713 | 1560 | 1980 |  | 264 | 209 | 308 |
| 12 | 1013 | 1360 | 1406 |  | 2700 | 2506 | 2036 |
| 17 | 20 | 36 | 40 |  | 522 | 509 | 472 |
| 18 | 7738 | 7065 | 6980 |  | 12642 | 16301 | 13690 |
| 35 | 78 | 89 | 98 |  | 1207 | 1309 | 1107 |
| 40 | 813 | 897 | 706 |  | 2059 | 2698 | 2560 |
| 44 | 1694 | 1506 | 1498 |  | 3618 | 3209 | 3019 |
| 50 | 516 | 560 | 590 |  | 1068 | 1598 | 1678 |
| 63 | 809 | 706 | 716 |  | 944 | 989 | 1022 |
| 70 | 4959 | 4602 | 4903 |  | 39 | 59 | 69 |
| 76 | 2831 | 2609 | 2860 |  | 654 | 603 | 598 |
| 88 | 12429 | 10369 | 13621 |  | 3042 | 3569 | 3097 |
| 92 | 4297 | 4698 | 4987 |  | 2033 | 1892 | 2569 |
| 2 | 23.2 | 50.9 | 60.3 |  | 571 | 509 | 546 |
| 6 | 215 | 269 | 198 |  | 600 | 629 | 687 |
| 10 | 370 | 309 | 356 |  | 1703 | 1986 | 1260 |
| 14 | 108 | 159 | 210 |  | 674 | 609 | 698 |
| 16 | 74 | 50 | 69 |  | 3439 | 3012 | 2860 |
| 19 | 1749 | 1069 | 2103 |  | 3358 | 3021 | 3690 |
| 22 | 4353 | 4010 | 3875 |  | 4532 | 4987 | 5069 |
| 26 | 171 | 226 | 259 |  | 3457 | 3018 | 3980 |
| 28 | 299 | 336 | 387 |  | 803 | 890 | 964 |
| 29 | 121 | 109 | 198 |  | 425 | 498 | 560 |
| 30 | 73 | 98 | 116 |  | 2918 | 2098 | 2510 |
| 45 | 2452 | 2069 | 2984 |  | 5906 | 6152 | 6589 |
| 59 | 2883 | 2609 | 2036 |  | 5064 | 5695 | 5369 |
| 80 | 1497 | 1056 | 987 |  | 65 | 96 | 69 |
| 5 | 250 | 209 | 298 |  | 651 | 601 | 789 |
| 31 | 391 | 349 | 456 |  | 2943 | 2690 | 3165 |
| 37 | 579 | 509 | 619 |  | 2799 | 2069 | 3069 |
| 39 | 142 | 193 | 215 |  | 1867 | 1603 | 2106 |
| 43 | 414 | 459 | 506 |  | 2050 | 2690 | 2730 |
| 56 | 186 | 256 | 290 |  | 2059 | 2651 | 1830 |
| 90 | 489 | 409 | 548 |  | 239 | 180 | 203 |
| 91 | 450 | 493 | 526 |  | 145 | 106 | 203 |
| 15 | 51 | 98 | 76 |  | 989 | 903 | 846 |
| 20 | 1455 | 1960 | 2106 |  | 8105 | 8360 | 7620 |
| 38 | 643 | 698 | 725 |  | 3752 | 3987 | 4120 |
| 41 | 1773 | 1609 | 2106 |  | 3240 | 3960 | 4102 |
| 49 | 297 | 336 | 206 |  | 1643 | 2106 | 1236 |
| 51 | 2378 | 2001 | 2987 |  | 6387 | 6698 | 7023 |
| 60 | 1155 | 906 | 860 |  | 1463 | 1560 | 1987 |
| 62 | 1370 | 1023 | 908 |  | 1455 | 1690 | 1560 |
| 78 | 3804 | 3206 | 4123 |  | 1643 | 1894 | 2016 |
| 85 | 5053 | 5560 | 4596 |  | 1995 | 1605 | 2105 |
| 89 | 13195 | 14985 | 19632 |  | 6018 | 6560 | 7012 |
| 9 | 23.6 | 36.9 | 40.6 |  | 77 | 90 | 106 |
| 21 | 95 | 162 | 149 |  | 620 | 687 | 702 |
| 25 | 3615 | 3087 | 4106 |  | 5311 | 5648 | 5930 |
| 52 | 587 | 635 | 694 |  | 2039 | 2698 | 3984 |
| 66 | 4554 | 4069 | 4987 |  | 1667 | 1987 | 2036 |
| 67 | 80076 | 81236 | 86954 |  | 17668 | 18975 | 19874 |
| 71 | 12084 | 13987 | 15069 |  | 4869 | 5126 | 5632 |
| 72 | 3772 | 3987 | 4120 |  | 1481 | 1687 | 1987 |
| 74 | 6664 | 6203 | 6987 |  | 1189 | 1029 | 1654 |
| 77 | 1686 | 1063 | 2016 |  | 552 | 598 | 609 |
| 81 | 4150 | 3269 | 3015 |  | 47 | 98 | 64 |
| 82 | 2196 | 2654 | 2987 |  | 89 | 109 | 126 |
| 83 | 1635 | 1987 | 2065 |  | 444 | 656 | 879 |
| 86 | 2742 | 2065 | 3012 |  | 141 | 198 | 210 |
