# Supplementary material for: Comparative Proteomic Analysis of Differentially Expressed Proteins Induced by Hydrogen Sulfide in Spinacia oleracea Leaves
Source: PLoS One. 2014 Sep 2;9(9):e105400. doi: 10.1371/journal.pone.0105400 (PMC4152154; doi:10.1371/journal.pone.0105400)
Supplement: Table S2 — Details of identified proteins (92) and peptides list of each protein in Spinacia oleracea leaves after treatment with 100 µM NaHS. (DOC) [file pone.0105400.s003.doc]

**Table S2**. Details of identified proteins (92) and peptides list of each protein in *Spinacia oleracea* leaves after treatment with 100 μM NaHS.

| **Spot^a^** | **NCBI  accession^b^** | **Protein identity^c^** | **Peptide sequence** |
| --- | --- | --- | --- |
| **Amino acid, nitrogen and sulfur metabolism** | | | |
| 42 | gi|108862760 | Glutathione synthetase, chloroplast precursor, putative, expressed | K.VVMNEQSGYLMR.T + 2 Oxidation (M) |
|  |  |  | K.EGGDALAAYILMQR.I + Oxidation (M) |
|  |  |  | K.ESYPFMLFLSSTWSK.H + Oxidation (M) |
|  |  |  | R.EGGGNNIYGIDVRETLIR.L |
|  |  |  | K.ESYPFMLFLSSTWSKHK.I + Oxidation (M) |
|  |  |  | K.VSSSDEGGVAAGFAVLDSLYLTDKAM.-  K.SAIQKPELFVLKPQREGGGNNIYGIDVR.E |
|  |  |  | R.ADPRSGTVPGVGLVHAPFSLLPTHLPESHWR.Q |
| 58 | gi|30683408 | Class I glutamine amidotransferase domain-containing protein | -.MITVLR.R |
|  |  |  | K.SHIEHR.V |
|  |  |  | K.KQDSDGR.L |
|  |  |  | K.SLENMVK.K |
|  |  |  | K.SLENMVK.K + Oxidation (M) |
|  |  |  | K.ATTHPVVSDKLSDK.S |
|  |  |  | K.LAATCATAVESRVQIDGR.I |
|  |  |  | K.LVAEVLLDEVAEKSFDLIVLPGGLNGAQR.F  K.MVADTLLSDITDSVFDLIVLPGGLPGGETLK.N + Oxidation (M) |
| 75 | gi|195651721 | Cysteine sulfinate desulfinase/cysteine desulfurase and related enzymes | R.GAAVLGCALGARK.Q |
|  |  |  | K.QPPESQELLSK.V |
|  |  |  | R.LGGFMPCSSAAR.S + Oxidation (M) |
|  |  |  | R.RDGHEGDVSNQGR.D |
|  |  |  | K.NHIFTSGASGTNAAVIR.G  K.NHIFTSGASGTNAAVIRGALR.A  R.TPMPTHLRLGGFMPCSSAAR.S  K.IPTQVETLVKGTAAVAEPEYK.Q  R.LCNMDIISKVQQVICFAFHDSK.L |
| **C-compound and carbohydrate metabolism** | | |  |
| 13 | gi|219810303 | Cellulose synthase CesA10 | R.LHQMR.N |
|  |  |  | R.LKGCAR.V |
|  |  |  | R.NELVVIR.R |
|  |  |  | K.ERMESWK.Q + Oxidation (M) |
|  |  |  | R.DGDPGPKPLR.Q |
|  |  |  | R.NDSQYVAESMLHAHMSYGR.G |
|  |  |  | K.GCARVPGDEEEDGVDDLENEFNWR.D |
|  |  |  |  |
| 27 | gi|207059706 | Caffeoyl CoA O-methyltransferase | R.LVDLVK.V |
|  |  |  | R.EPTPMK.E |
|  |  |  | M.AINEETK.I |
|  |  |  | K.HPMNVMATSADEGQFLNMLLK.L + 3 Oxidation (M) |
|  |  |  | K.VGGLIGYDNTLWNGPVAAPPDAPMAK.Y |
|  |  |  | K.HPMNVMATSADEGQFLNMLLKLVNAK.N |
|  |  |  | R.EITAKHPMNVMATSADEGQFLNMLLK.L + 3 Oxidation (M) |
|  |  |  |  |
| 57 | gi|12322095 | Trehalose-phosphatase, putative | R.LTQGRK.V |
|  |  |  | K.GSIGVAVR.V |
|  |  |  | R.IHTWIEAMR.A |
|  |  |  | R.IHTWIEAMRASSPTR.T |
|  |  |  | K.GVLFQAANEFLPMIDK.V |
|  |  |  | M.TNHNALISDAKGSIGVAVR.V |
|  |  |  | K.QIVMFLDYDGTLSPIVENPDR.A |
|  |  |  |  |
| 64 | gi|145408196 | Secondary wall-associated glycosyltransferase family 8D | K.GCHIRPS.- |
|  |  |  | K.TFAVKLR.E |
|  |  |  | K.GVREFIK.V |
|  |  |  | R.GEDKFVMSK.K |
|  |  |  | K.VPVMEAMEK.D + Oxidation (M) |
|  |  |  | K.IQEYLYRHVASSSIPK.Q |
|  |  |  | K.GRSDIPQTLEEFMDEVK.N |
|  |  |  | R.AKPWLDIAFPQLRPLWAK.Y  K.QLHCLALRLASEHSTNAAAR.L  R.FVFVLSTVDSIDGETKCSTLGCLGER.L |
| 73 | gi|170102 | Carbonic anhydrase precursor | K.FMVFACSDSR.V |
|  |  |  | K.FMVFACSDSR.V + Oxidation (M) |
|  |  |  | R.NIANMVPVFDKDK.Y |
|  |  |  | K.YAGVGAAIEYAVLHLK.V |
|  |  |  | K.GQAPKFMVFACSDSR.V + Oxidation (M) |
|  |  |  | K.EAVNVSLGNLLTYPFVR.D  R.VCPSHVLDFQPGEAFMVR.N  R.VCPSHVLDFQPGEAFMVR.N + Oxidation (M)  K.VLAEHGNATFAEQCTHCEK.E  K.VAQITSELADGGTPSASYPVQR.I  R.VCPSHVLDFQPGEAFMVRNIANMVPVFDK.D + Oxidation (M) |
| 87 | gi|302811518 | Quasimodo1-like protein | K.DATNMK.F |
|  |  |  | R.DLRER.L |
|  |  |  | K.MENATK.D |
|  |  |  | K.AHIEVK.A |
|  |  |  | K.RTILALR.S |
|  |  |  | K.MENATKDATNMK.F |
|  |  |  | K.YMNFSHPLIASR.F |
|  |  |  | R.NYTELLARPENR.G |
|  |  |  | R.FYFENKMENATK.D + Oxidation (M)  K.SLHCLTMRLMEER.V + 2 Oxidation (M)  K.QAKDQLAVVNAYAAYAR.R  R.YVDYDMELVQMCNFGH.- + Oxidation (M)  K.LGTLPPGLITFYKTTKPLDK.S  K.DLTGLWEIDMDGKVNGAVETCFGSFHR.Y |
| **Phosphate metabolism** | |  |  |
| 4 | gi|25137409 | S-locus receptor kinase | K.EGSQGQR.S |
|  |  |  | K.SVWSTNLTR.G |
|  |  |  | R.SIGMKECEK.R |
|  |  |  | K.LPDTMMAIVDR.S + Oxidation (M) |
|  |  |  | R.VTNNSFYSRLK.I  K.TTLSSRWYLGMWYK.K + Oxidation (M) |
|  |  |  | K.LPDTMMAIVDRSIGMK.E + 2 Oxidation (M) |
| 32 | gi|81075765 | Ser/Thr protein kinase-like | R.TIGVCHR.D |
|  |  |  | K.NVHAEILLK.L |
|  |  |  | R.LPEEMNDMR.I + Oxidation (M) |
|  |  |  | R.LLDHPNVVSLK.H |
|  |  |  | R.LPEEMNDMRIR.D + 2 Oxidation (M) |
|  |  |  | R.NGQPKQTISYMAER.V + Oxidation (M) |
|  |  |  | R.LPNGRFLPPLFNFK.A |
|  |  |  | K.NVHAEILLKLVPEHAR.K  R.MPLILVKLYTYQIFR.A + Oxidation (M) |
| 79 | gi|179399401 | Putative calcium dependent protein kinase | K.YSRMK.Q |
|  |  |  | R.MHTGLK.G |
|  |  |  | K.EDSPLK.A |
|  |  |  | K.YSRMK.Q + Oxidation (M) |
|  |  |  | R.NKPDFR.R |
|  |  |  | R.IVASPSCA.- |
|  |  |  | R.LLRSASMSSR.I |
|  |  |  | K.GSMEPLLEEADIDKDGR.I + Oxidation (M) |
|  |  |  | K.SGPESDVWSIGVITYILLCGR.R |
|  |  |  | K.VAAECHLHGLVHRDMKPENFLFK.S |
|  |  |  | R.KPSTTQLTTSGVPSRTSGVPTTTITHYHNDK.H |
|  |  |  |  |
| **Energy production and photosynthesis** | | | |
| 1 | gi|170129 | Rubisco activase precursor | K.KLVNSK.D |
|  |  |  | K.NYDPTAR.S |
|  |  |  | K.FYWAPTR.E |
|  |  |  | K.NFLNLPNIK.I |
|  |  |  | K.IPLILGVWGGK.G |
|  |  |  | K.DFSDDQLDIR.R |
|  |  |  | K.FYWAPTREDR.I |
|  |  |  | K.DFSDDQLDIRR.G |
|  |  |  | K.DGPPVFEQPEMTLQK.L |
|  |  |  | K.LVDAFPGQSIDFFGALR.A |
|  |  |  | K.LMEYGNMLVQEQENVKR.V |
|  |  |  | R.VPIIVTGNDFSTLYAPLIR.D |
|  |  |  | K.YDIDNMLGDFYIAPAFMDK.L |
|  |  |  | R.VQLADQYMSSAALGDANKDAIDR.G |
|  |  |  |  |
| 3 | gi|306481796 | Ribulose-1,5-bisphosphate carboxylase/oxygenase large subunit | R.EACKW.- |
|  |  |  | K.ALRALR.L |
|  |  |  | K.DTDTLAAFR.V |
|  |  |  | R.EITLGFVDLLR.D |
|  |  |  | R.MSGGDHIHAGTVVGK.L |
|  |  |  | K.GHYLNATAGTCEEMIK.R |
|  |  |  |  |
| 7 | gi|54303888 | Ribulose-1,5-bisphosphate carboxylase/oxygenase large subunit | K.DTDILAAFR.V |
|  |  |  | R.FVFCAEAIYK.A |
|  |  |  | K.DDENVNSQPFMR.W |
|  |  |  | R.MSGGDHIHAGTVVGK.L |
|  |  |  | K.TFQGPPHGIQVERDK.L |
|  |  |  | R.GGLDFTKDDENVNSQPFMR.W |
|  |  |  | K.AQAETGEIKGHYLNATAATCEEMIK.R |
|  |  |  |  |
| 8 | gi|49182654 | Ribulose-1,5-bisphosphate carboxylase/oxygenase large subunit | K.ALRALR.L |
|  |  |  | K.RAACAR.E |
|  |  |  | R.IPPSYVK.T |
|  |  |  | K.NHGMHFR.V |
|  |  |  | K.TFQGPPHGIQVER.D |
|  |  |  | K.ALRLSGGDHIHAGTVVGK.L |
|  |  |  | K.DDENVNSQPFMRWR.D + Oxidation (M) |
|  |  |  | R.GGLDFTKDDENVNSQPFMR.W |
|  |  |  | R.ELGVPIVMHDYLTGGFTANTTLAHYR.R |
|  |  |  |  |
| 11 | gi|15235029 | Chlorophyll a-b binding protein CP26 | K.WYGPDR.R |
|  |  |  | K.KPAPAKSK.A |
|  |  |  | K.WYGPDRR.I |
|  |  |  | R.IFLPDGLLDR.S |
|  |  |  | K.YQAFELIHAR.W |
|  |  |  | K.LHPGGPFDPLGLAK.D |
|  |  |  | K.TGALLLDGNTLNYFGK.N |
|  |  |  | R.ITNGLDFEDKLHPGGPFDPLGLAK.D |
|  |  |  | K.NIPINLVLAVVAEVVLLGGAEYYR.I |
|  |  |  |  |
| 23 | gi|255549948 | Photosystem I reaction center subunit VI, chloroplast precursor, putative | R.NGAVEAK.Y |
|  |  |  | K.RGLLLK.F |
|  |  |  | K.GLGGSSLTGTKLHVKPSR.Q |
|  |  |  | M.ASLATFAAVQPATVKGLGGSSLTGTK.L |
|  |  |  | K.FLILGGGSTLAYLSATATGDVLPIKK.G |
|  |  |  |  |
| 34 | gi|170129 | Rubisco activase precursor | K.WAHLAK.D |
|  |  |  | R.VYDDEVR.K |
|  |  |  | K.FYWAPTR.E |
|  |  |  | K.NFLNLPNIK.I |
|  |  |  | K.IPLILGVWGGK.G |
|  |  |  | K.DFSDDQLDIR.R |
|  |  |  | K.SFQCELVFAK.L |
|  |  |  | K.FYWAPTREDR.I |
|  |  |  | K.DGPPVFEQPEMTLQK.L |
|  |  |  |  |
| 36 | gi|255551591 | NADH dehydrogenase, putative | K.DLHHR.C |
|  |  |  | K.GQQVTR.C |
|  |  |  | K.DPNPEK.C |
|  |  |  | K.KDPNPEK.C |
|  |  |  | R.CFQENVDFLK.C |
|  |  |  | M.ASAVDTTGNPIPTSAVLTASSK.H |
|  |  |  | K.EMDAYVGCMYYHTNEFDLCR.K |
|  |  |  | K.EMDAYVGCMYYHTNEFDLCRK.E + 2 Oxidation (M) |
|  |  |  |  |
| 46 | gi|131392 | Oxygen-evolving enhancer protein 2, chloroplastic; | K.AQAGDKR.W |
|  |  |  | K.EFPGQVLR.Y |
|  |  |  | K.HQVIAATVK.D |
|  |  |  | K.EKEFPGQVLR.Y |
|  |  |  | K.QYYSITVLTR.T |
|  |  |  | K.NTEFMPYNGDGFK.L |
|  |  |  | R.YQAVSIKPNQIVCK.A |
|  |  |  | R.YEDNFDATSNLSVLVQPTDK.K |
|  |  |  | K.SITDFGSPEDFLSQVDYLLGK.Q |
|  |  |  | R.YEDNFDATSNLSVLVQPTDKK.S |
|  |  |  | K.TDSEGGFDSGVVASANVLESSTPVVDGK.Q |
|  |  |  |  |
| 47 | gi|755801 | ATP synthase | K.GNTYFIR.R |
|  |  |  | K.TLSINYNR.A |
|  |  |  | R.RPEIPVDR.Y |
|  |  |  | K.KGNTYFIR.R |
|  |  |  | K.VALMVVTGDR.G |
|  |  |  | R.YFDGTNLPTAK.E |
|  |  |  | R.GLCGGFNNMLLK.K |
|  |  |  | K.CVDAAEDELFR.L |
|  |  |  | R.ALQESLASELAAR.M |
|  |  |  | K.SDPVIHTLLPLSPK.G |
|  |  |  | K.EAQAIADDVFSLFVSEEVDKVEMLYTK.F |
|  |  |  |  |
| 48 | gi|297842481 | Thylakoid lumenal 29.8 kDa protein | K.ISAHHR.V |
|  |  |  | R.CATALSR.R |
|  |  |  | R.VDGETQR.D |
|  |  |  | K.KISAHHR.V |
|  |  |  | -.METALLR.Y + Oxidation (M) |
|  |  |  | R.IASSFRIV.- |
|  |  |  | M.ETALLRYCFNFSSHPYHK.K |
|  |  |  | K.IENIGPPAKVINAFGPEVIGENVEGK.V |
|  |  |  |  |
| 53 | gi|298570223 | Ribulose-1,5-bisphosphate carboxylase/oxygenase large subunit | R.IPPSYTK.T |
|  |  |  | R.QKNHGMHFR.V + Oxidation (M) |
|  |  |  | R.FLFCAEALYK.A |
|  |  |  | K.DDENVNSQPFMR.W |
|  |  |  | K.WSPELAAACEVWK.E |
|  |  |  | K.TFQGPPHGIQVERDK.L |
|  |  |  | K.EYKLTYYTPEYETK.D |
|  |  |  | R.ESTLGFVDLLRDDYIEK.D |
|  |  |  | R.ELGVPIVMHDYLTGGFTANTSLAHYCR.D + Oxidation (M) |
|  |  |  |  |
| 54 | gi|2392029 | Chain L, activated spinach rubisco in complex with the product 3- phosphoglycerate | R.AMHAVIDR.Q + Oxidation (M) |
|  |  |  | R.NEGRDLAR.E |
|  |  |  | R.VALEACVQAR.N |
|  |  |  | R.QKNHGMHFR.V + Oxidation (M) |
|  |  |  | R.DITLGFVDLLR.D |
|  |  |  | K.WSPELAAACEVWK.E |
|  |  |  | K.TFQGPPHGIQVERDK.L |
|  |  |  | K.LEGERDITLGFVDLLR.D |
|  |  |  | R.DITLGFVDLLRDDYTEK.D |
|  |  |  | R.ELGVPIVMHDYLTGGFTANTTLSHYCR.D |
|  |  |  |  |
| 55 | gi|307548298 | Phosphoenolpyruvate carboxylase | R.ECHVR.D |
|  |  |  | R.AIQAAFR.T |
|  |  |  | R.CNDELR.A |
|  |  |  | R.CNDELRAR.A |
|  |  |  | R.LPYNVPLIQFCSWMGGDR.D |
|  |  |  | K.LMDEMAVVATEEYRSIVFR.E + 2 Oxidation (M) |
|  |  |  | K.GDPSIAGLYDELLVADDLKPFGEQLR.D |
|  |  |  | R.VTVQGEVIEHSFGEEHLCFRTLER.F |
|  |  |  | K.NIGIDERLPYNVPLIQFCSWMGGDR.D |
|  |  |  |  |
| 61 | gi|131392 | RecName: Full=Oxygen-evolving enhancer protein 2, chloroplastic; | K.AQAGDKR.W |
|  |  |  | K.EFPGQVLR.Y |
|  |  |  | K.EKEFPGQVLR.Y |
|  |  |  | K.QYYSITVLTR.T |
|  |  |  | K.KNTEFMPYNGDGFK.L |
|  |  |  | R.TADGDEGGKHQVIAATVK.D |
|  |  |  | K.VSPADAAYGEAANVFGKPK.K |
|  |  |  | R.YEDNFDATSNLSVLVQPTDKK.S |
|  |  |  |  |
| **Lipid, fatty acid and isoprenoid metablism** | | |  |
| 24 | gi|226496803 | Serine palmitoyltransferase 2 | R.VDGGTTK.L |
|  |  |  | R.VIESLK.K |
|  |  |  | R.ECLRQK.V |
|  |  |  | K.FVDWFK.A |
|  |  |  | K.EIIQHLK.H |
|  |  |  | K.VAVVTVAFPATPLLLAR.A |
|  |  |  | R.QKVAVVTVAFPATPLLLAR.A |
|  |  |  | R.GVCELLGVDPADVDIMMGTFTK.S + Oxidation (M) |
|  |  |  |  |
| 33 | gi|194067759 | Adenylate isopentenyltransferase | K.MQVYK.G + Oxidation (M) |
|  |  |  | R.MRQGVLQTGIDNIK.A |
|  |  |  | K.ANTCKLASCQLQNILR.L |
|  |  |  | R.AIGVPEMDQYFRILEASDER.M + Oxidation (M) |
|  |  |  | M.MSCCFCKQTQPMVSFPSGLNISSR.W + Oxidation (M) |
|  |  |  | K.LASCQLQNILRLQSQLEGWNIVHR.L |
|  |  |  |  |
| 65 | gi|255594379 | Acyl-CoA dehydrogenase, putative | K.VIDPLK.Q |
|  |  |  | R.KVIDPLK.Q |
|  |  |  | K.ETGSFVASL.- |
|  |  |  | K.GFELAQVR.L |
|  |  |  | R.SVHIHGALGCSDEMPLAR.L + Oxidation (M) |
|  |  |  | K.FFSSNINNASFTIVMTITNPDVSVHK.G |
|  |  |  | K.HGVKILPADSEHSALFQCMQGLPEGALR.R + Oxidation (M) |
|  |  |  |  |
| 68 | gi|209402461 | Putative plastid 1-deoxy-D-xylulose 5-phosphate reductoisomerase precursor | R.DTTLAEMR.D + Oxidation (M) |
|  |  |  | K.MGDLTFREPDVEK.Y + Oxidation (M) |
|  |  |  | R.ADSNNESRYGACWPGR.A |
|  |  |  | K.FEIVALSAGKNLAVMAEQIK.K + Oxidation (M) |
|  |  |  | K.IGYFDIYRTIEMAMEAHK.N + 2 Oxidation (M) |
|  |  |  | R.AGGTMTGVFSAANEQAVAMFLDKK.I |
|  |  |  | K.DLIKDMDGDMPEILYGADGMVEVAR.H + 3 Oxidation (M) |
|  |  |  | K.HGVKILPADSEHSALFQCMQGLPEGALR.R + Oxidation (M) |
|  |  |  |  |
| 69 | gi|297847516 | Lipase class 3 family protein | R.REIIR.Y |
|  |  |  | K.DEEAQK.R |
|  |  |  | R.CDELGVK.V |
|  |  |  | K.SCDFLRSEYHVPPCWR.Q |
|  |  |  | R.YGEFAQACYDSFDFDPHSK.Y |
|  |  |  | K.TSFPWSYAHVGVELALDHKK.S |
|  |  |  | R.EVQGCNNWEGLLDPMNNHLR.R |
|  |  |  | K.FKTCSIICASSCTSISSSTTQQK.Q |
|  |  |  | R.YGEFAQACYDSFDFDPHSKYCGSCK.Y |
|  |  |  |  |
| 84 | gi|30687094 | Cyclopropane-fatty-acyl-phospholipid synthase | R.ITFELR.D |
|  |  |  | R.KNFLER.Q |
|  |  |  | K.DSTCPLK.S |
|  |  |  | K.SDDEDLR.T |
|  |  |  | K.EVVLYEK.E |
|  |  |  | K.YFLKHVSR.Q |
|  |  |  | K.EDVLNYIEK.L |
|  |  |  | K.DSTCPLKSILK.I |
|  |  |  | R.HMVPSLTETGARLFVTR.F |
|  |  |  |  |
| **Transcription, protein synthesis, folding, modification, destination** | | |  |
| 12 | gi|30692594 | Putative F-box/LRR-repeat protein 9 | R.GVVEAAMK.L |
|  |  |  | -.MSSSSSSPFPQAMK.N + Oxidation (M) |
|  |  |  | R.EQELKVVGQSCPNLR.T |
|  |  |  | R.NGLSETGLNAILEGCPHLK.N |
|  |  |  | R.LGVVDCAPVLSRGVVEAAMK.L + Oxidation (M) |
|  |  |  | R.IKENLVNSVELFYVIEPLCCR.A |
|  |  |  |  |
| 17 | gi|334183835 | Small subunit ribosomal protein S1 | R.IAWHRVR.Q |
|  |  |  | K.VFTEAEEMAK.K + Oxidation (M) |
|  |  |  | R.SGLLHISNITR.R |
|  |  |  | K.DDDEGVEIAEFAR.Q |
|  |  |  | R.FLVQITRLNEDK.N |
|  |  |  | R.IGSVSDVLQVDESVK.V |
|  |  |  | K.LDVNIGADMLGTMLTK.E + Oxidation (M) |
|  |  |  | K.MGIVKDDDEGVEIAEFAR.Q |
|  |  |  | K.SLFPDKISLSIADLESEPGLFISDR.E |
|  |  |  | -.MQTLLCQPCKSLPILTASSSSSLIR.S + Oxidation (M) |
|  |  |  |  |
| 18 | gi|170131 | Ribosomal protein 30S subunit | K.VGKSVAK.H |
|  |  |  | K.LILQGR.N |
|  |  |  | R.HMKGFNR.S |
|  |  |  | R.CEVTLFTK.R |
|  |  |  | R.LSARGGDLSK.G |
|  |  |  | R.NLEVSDNVR.S |
|  |  |  | R.AEEDAESLYSSIDLVSSIIQR.K |
|  |  |  | R.EEVLEEVESAPAPVSVEDDDFIEEVVR.T |
|  |  |  | K.YFDMPPLTITEAVEQLENVDHDFYAFR.N |
|  |  |  | K.YFDMPPLTITEAVEQLENVDHDFYAFR.N + Oxidation (M) |
|  |  |  |  |
| 35 | gi|255961421 | Ribosomal protein L22 | R.VLDEIR.W |
|  |  |  | K.ANLFITK.A |
|  |  |  | R.IHMSVFK.A |
|  |  |  | K.TMCHITIVLNIVK.K + Oxidation (M) |
|  |  |  | K.TMCHITIVLNIVKK.S |
|  |  |  | R.WRYYEETVMILNLMPYQASYPILK.L + 2 Oxidation (M) |
|  |  |  |  |
| 40 | gi|159470805 | Peptidyl-prolyl cis-trans isomerase, FKBP-type | M.MLNQR.T + Oxidation (M) |
|  |  |  | R.CTIVAR.A |
|  |  |  | R.IQTQNR.V |
|  |  |  | K.TMKPGGKR.R + Oxidation (M) |
|  |  |  | R.QVMMFSDLVCE.- + Oxidation (M) |
|  |  |  | R.RQVMMFSDLVCE.- + Oxidation (M) |
|  |  |  | R.CTIVARAGAGPSGQPLQR.A |
|  |  |  | R.RIIVPPALGPPVGPSTFFSAK.Q |
|  |  |  | R.EGFDVKVLGEGYQVAPSGLIYK.D |
|  |  |  |  |
| 44 | gi|255582427 | Threonyl-tRNA synthetase, putative | R.FVSLSK.L |
|  |  |  | K.VYPEAK.V |
|  |  |  | R.NAEKQK.I |
|  |  |  | R.EVTSKLK.E |
|  |  |  | K.LKENGIR.T |
|  |  |  | K.RFVSLSK.L |
|  |  |  | R.ENMYDQMK.I + 2 Oxidation (M) |
|  |  |  | R.HIIEDSWKK.I |
|  |  |  | R.YELSGSLHGLFRVR.G |
|  |  |  | R.FSGELGTMTIDDFISR.I |
|  |  |  | R.IMAVNEPYKMEILDSIK.E |
|  |  |  | R.VAELGTVYRYELSGSLHGLFR.V |
|  |  |  | R.NGLSISTAVATTESAPVAQNDIAEDTQK.E |
|  |  |  | K.LFSLQDLLALMHGDVEIVLLNHNCSLK.F + Oxidation (M) |
|  |  |  |  |
| 50 | gi|77556384 | F-box domain containing protein | R.HADWR.G |
|  |  |  | R.NLHMR.D + Oxidation (M) |
|  |  |  | R.WCAALR.R |
|  |  |  | -.MDNVRR.S + Oxidation (M) |
|  |  |  | R.VLHPESEIR.E |
|  |  |  | R.GDLVSFVAADASGR.C |
|  |  |  | R.HLGLRVNYTAMVDMLPR.Q + Oxidation (M) |
|  |  |  | R.ALLDGTSLVALHLFYFTVEAYHIDR.L |
|  |  |  | R.DGGGGDVSSDDEEYGIYDDVTANDDGGYEIGR.V |
|  |  |  |  |
| 63 | gi|302379151 | PRP-like protein | R.TDGLDKK.N |
|  |  |  | K.IENHLSVVPNADGGSTTK.T |
|  |  |  | K.YAEAQNTMLFKAVEAYLIAN.- + Oxidation (M) |
|  |  |  | K.NCTIDYSYIDGDILMGFIDK.I + Oxidation (M) |
|  |  |  | K.IENHLSVVPNADGGSTTKTTAIFHTK.G |
|  |  |  |  |
| 70 | gi|255539022 | Skp1, putative | K.HVETSK.S |
|  |  |  | K.TPEEIR.K |
|  |  |  | K.VIEYCK.K |
|  |  |  | K.GKTPEEIR.K |
|  |  |  | K.NDFTPEEEEEVR.R |
|  |  |  | K.NDFTPEEEEEVRR.E |
|  |  |  | K.ITLKSSDGETFEVDEAVALESQTIK.H |
|  |  |  |  |
| 76 | gi|14150732 | Hypersensitive-induced response protein | R.AEGDAESK.Y |
|  |  |  | R.VAANEKAEAEK.I |
|  |  |  | K.ASDAFYRLSNTR.E |
|  |  |  | K.TKDNVFVNVVASVQYR.A |
|  |  |  | -.MGQALGLVQVDQSTVAIK.E + Oxidation (M) |
|  |  |  | K.DVMDMVLVTQYFDTMK.E + Oxidation (M) |
|  |  |  | K.SSSVFIPHGPGAVKDIAAQIR.D |
|  |  |  | K.AMSTYGYEIVQTLIVDIEPDEHVK.R + Oxidation (M) |
|  |  |  |  |
| 88 | gi|55296320 | Putative DNA-(apurinic or apyrimidinic site) lyase | R.EHDHEGR.V |
|  |  |  | R.VKPISVQYGIGIR.E |
|  |  |  | R.VNNWDPCFSNYVK.I |
|  |  |  | K.ILSWNINGLHDVVTTK.G |
|  |  |  | K.NLIADYDSYWSCSVSR.L |
|  |  |  | K.CMKILSWNINGLHDVVTTK.G |
|  |  |  | K.VHDSYILPDVSFSDHSPIGLVLK.L |
|  |  |  | R.LPEWFAYNPKTMRPPPLSHDTK.C + Oxidation (M) |
|  |  |  | K.VHDSYILPDVSFSDHSPIGLVLKL.- |
|  |  |  |  |
| 92 | gi|15222035 | Two-component response regulator ARR15 | K.ELIMR.G + Oxidation (M) |
|  |  |  | R.GGEAEEGK.T |
|  |  |  | K.IKESSALR.E |
|  |  |  | K.ELIMRGGEAEEGK.T + Oxidation (M) |
|  |  |  | K.ISACKVTTVESGTR.A |
|  |  |  | R.ALQYLGLDGDNGSSGLK.D |
|  |  |  | R.EIPVVIMSSENIQPR.I |
|  |  |  | R.IEQCMIEGAEEFLLKPVK.L + Oxidation (M) |
|  |  |  | R.IEQCMIEGAEEFLLKPVKLADVK.R |
| **Cell rescue, development and defense** | | |  |
| 2 | gi|238814300 | Pollen coat-like protein | K.ESMQEA.- |
|  |  |  | K.GQAQQKGNQMMDK.A + Oxidation (M) |
|  |  |  | K.GNQMMDKASNAAQSPK.E + 2 Oxidation (M) |
|  |  |  | -.MDSSQNMSYQAGQTKGQAQQK.G + Oxidation (M) |
|  |  |  | -.MDSSQNMSYQAGQTKGQAQQK.G + 2 Oxidation (M) |
|  |  |  |  |
| 6 | gi|357490825 | NBS-LRR resistance protein | R.DVSMHREYR.E |
|  |  |  | K.LLEAFELAALCFR.K |
|  |  |  | R.CDGFRFLMCDGCR.G + Oxidation (M) |
|  |  |  | R.KDGNSIPPVISVVEYR.R |
|  |  |  | R.RISAAETEVLPPVLFVK.G |
|  |  |  | R.ISAAETEVLPPVLFVKGR.C |
|  |  |  | R.VNDEDDIVFPEEEEIR.K |
|  |  |  |  |
| 10 | gi|39841264 | Phl p 3 allergen | K.KGNVWEVK.S |
|  |  |  | R.QHGSEEWEPLTKK.G |
|  |  |  | K.GGMRNVFDEVIPTAFSIGK.T |
|  |  |  | K.LVLDIKYTRPGDSLAEVELR.Q |
|  |  |  | R.NVFDEVIPTAFSIGKTYKPEE.- |
|  |  |  |  |
| 14 | gi|626032 | Lipoxygenase | K.GNVYVPR.D |
|  |  |  | R.VKAVATIK.V |
|  |  |  | R.KNDLQQK.R |
|  |  |  | R.VMTVEEAINQK.R + Oxidation (M) |
|  |  |  | R.LLEHLRDTPAEK.I |
|  |  |  | R.GMAEEDPTAEQGLK.L |
|  |  |  | R.SLSLELCSSELDAKTGK.E |
|  |  |  | R.IFFANKTYLPGQTPAGLR.S |
|  |  |  | R.QEMFLEDINLTASDGAGNSTVLPIR.C |
|  |  |  |  |
| 16 | gi|302793903 | Allene oxide synthase | K.NFFEK.R |
|  |  |  | R.EEVRR.A |
|  |  |  | K.FKPRR.F |
|  |  |  | R.RFMGEEGQR.L |
|  |  |  | R.FYGKNFFEK.R |
|  |  |  | R.DPVVFEEPEK.F |
|  |  |  | R.DPVVFEEPEKFKPR.R |
|  |  |  | K.IKAGEMLYGYQPLVTR.D |
|  |  |  | K.NFVVLVTRILFADFFLHYDSFK.L |
|  |  |  |  |
| 19 | gi|224113557 | cc-nbs-lrr resistance protein | R.MEDIGNK.C |
|  |  |  | R.AVTDESVK.R |
|  |  |  | R.AVTDESVKR.W |
|  |  |  | K.RLIVDECK.L |
|  |  |  | R.FTDCKSLEK.L |
|  |  |  | K.EMQEWQSILK.S |
|  |  |  | R.GDDEAALTAVDARK.L |
|  |  |  | K.CRQLPTLGCLPR.L |
|  |  |  | R.TVFSMVDVFNGSWK.F + Oxidation (M) |
|  |  |  | R.LSSIVEFEISGCDELR.Y |
|  |  |  | R.DPDRETHSFLDSSEVVGR.E |
|  |  |  | K.VSGGGRETIAPDLESIGTEIAK.K |
|  |  |  | K.CIGNEFYSSSGSAAVLFPALK.K  R.ETHSFLDSSEVVGREGDVFK.V  K.ILGAMLQNIDKTTGGLSNLNAIMENLK.K |
| 22 | gi|1680686 | Rust resistance kinase Lr10 | K.VEMFLK.T + Oxidation (M) |
|  |  |  | R.YTFSEVKK.M |
|  |  |  | R.GGISLPHMLK.D + Oxidation (M) |
|  |  |  | K.VGQGGFGSVYK.G |
|  |  |  | R.DQSIVTLTAAR.G |
|  |  |  | K.TRYNAEIHMK.V + Oxidation (M) |
|  |  |  | R.GTMGYIAPELYSR.N + Oxidation (M) |
|  |  |  | R.ALIYEFMPNESLEK.Y |
|  |  |  | K.ADVYSFGMLVLEMVSGRR.N + 2 Oxidation (M) |
|  |  |  | K.YIFSDDSNIFQNLLVPEK.L |
|  |  |  | K.LACSGQDTILVHPVLGPYSVSAIDYR.R |
|  |  |  |  |
| 26 | gi|304325281 | Rp1-like protein | R.EDCFR.L |
|  |  |  | K.HLSIDVCR.S |
|  |  |  | K.VWMKENIIK.A + Oxidation (M) |
|  |  |  | R.SILWEDYSSLK.Q |
|  |  |  | R.YLNLIRTLVSELPR.S |
|  |  |  | R.LQRCFLYCSLFPK.G |
|  |  |  | R.EDCFRLEDDNVTEIPCTVR.H |
|  |  |  | R.LEDDNVTEIPCTVRHLSVHVQSMQK.H |
|  |  |  | K.SGKSLLLGEHGSSSTATTVTKPFHAAMSR.A + Oxidation (M) |
|  |  |  | R.FRVQESLTVSSSVLLNHMLMAEGFTVPPK.L |
|  |  |  |  |
| 28 | gi|168068013 | GLP5 GID1-like protein | -.MPGVGVK.L + Oxidation (M) |
|  |  |  | K.FLLKQR.L |
|  |  |  | R.MTIRDASSIEPVK.V |
|  |  |  | -.MPGVGVKLYSVFFK.F + Oxidation (M) |
|  |  |  | R.MAKACNVIVIAVGYR.L + Oxidation (M) |
|  |  |  | K.VVAQALMYPFFLGKVQTR.S + Oxidation (M) |
|  |  |  | R.DASSIEPVKVVAQALMYPFFLGK.V |
|  |  |  |  |
| 29 | gi|50252814 | Ethylene-forming enzyme-like | R.GSASTVR.A |
|  |  |  | R.KVILAR.Y |
|  |  |  | R.GSASMVR.A |
|  |  |  | R.YQVMIPNRYQR.V |
|  |  |  | K.ASSPVVPPSTSHAGARR.R |
|  |  |  | R.QPHPQPSAPHRSSSPAASSSVIHAPR.R |
|  |  |  | R.DIITEAAAMAFADPNLQIPDRYDR.S |
|  |  |  |  |
| 30 | gi|149939807 | RPM1-interacting protein 4 | K.VREER.S |
|  |  |  | M.ARSNVPK.F |
|  |  |  | R.ADESPEK.V |
|  |  |  | R.EHMRSR.E |
|  |  |  | K.NSYDGTGK.T |
|  |  |  | R.EERSSGANVSGSSR.T |
|  |  |  | R.SNVPKFGNWEAEENVPYTAYFDK.A |
|  |  |  |  |
| 45 | gi|15081223 | Glycine-rich protein GRP17 | K.GGLKAWCK.K |
|  |  |  | R.FFPSLSLSERDGR.K |
|  |  |  | R.EGRFFPSLSLSER.D |
|  |  |  | -.MSEELSQKPSSAQSLSLR.E + Oxidation (M) |
|  |  |  | K.GMSGGISGSEEGMSGSEGGMSSGGGSK.S + Oxidation (M) |
|  |  |  | K.GMSGGSESEEGMSGSEGGMSGGGGSKSK.S + Oxidation (M) |
|  |  |  | K.NCMSGGMSGSEEGMSGSEGGMSGGGGGK.S |
|  |  |  | K.KSMSGGMSGSEGGMSGSEGGMSGGGMSGGSGSK.H + Oxidation (M) |
|  |  |  |  |
| 59 | gi|156141675 | Putative NBS domain resistance protein | R.ELLRR.K |
|  |  |  | K.LAEHVR.E |
|  |  |  | R.EQLKTR.M |
|  |  |  | K.VAFPTNK.K |
|  |  |  | K.IEDWDK.L |
|  |  |  | K.KVLHDPK.I |
|  |  |  | K.LAEHVREQLK.T |
|  |  |  | R.MYLIVMDDVWK.I + Oxidation (M) |
|  |  |  |  |
| 80 | gi|15808946 | Auxin-regulated protein | R.CTNQAVK.D |
|  |  |  | R.IIESDCK.E |
|  |  |  | M.SRTTELQMHK.K + Oxidation (M) |
|  |  |  | R.TKVWTEPPNHK.L |
|  |  |  | R.IVEEEEELSENR.I |
|  |  |  | R.NGQLEHPHFMEVPLSSR.D + Oxidation (M) |
|  |  |  | K.SKAASVLMQLLYCGSMSFK.Q + 2 Oxidation (M) |
|  |  |  | K.VPVVYYLSRNGQLEHPHFMEVPLSSR.D |
|  |  |  |  |
| **Cellular transport, transport facilities, transport routes and cellular signal transduction** | | |  |
| 5 | gi|112145418 | WRKY transcription factor 23 | R.LGDMLR.A + Oxidation (M) |
|  |  |  | R.RLGDMLR.A + Oxidation (M) |
|  |  |  | R.ALVVKYSDLQGK.V |
|  |  |  | K.KLVEQMAATLTR.D |
|  |  |  | K.SHEVEALEAELR.R |
|  |  |  | R.GGGPPVPSKVLVEEDFMSSK.K |
|  |  |  | K.VSGMMAAAAANNHQSLTLTTSEGGSAASPSRK.R |
|  |  |  |  |
| 31 | gi|5834502 | Potassium channel | R.TMEFR.N + Oxidation (M) |
|  |  |  | K.AEYIPPR.E |
|  |  |  | K.TICKSIR.H |
|  |  |  | K.TICKSIR.H |
|  |  |  | K.RNELTVMK.E + Oxidation (M) |
|  |  |  | K.LGDLFHEVR.A |
|  |  |  | K.IITPMDSRYR.C + Oxidation (M) |
|  |  |  | K.GVSREILLLLVADMK.A |
|  |  |  | R.QEDNLIMIKNFLQHHK.K + Oxidation (M) |
|  |  |  | R.EILLLLVADMKAEYIPPR.E + Oxidation (M) |
|  |  |  |  |
| 37 | gi|63094976 | Phytochrome C | R.SHSAREIK.W |
|  |  |  | R.MICDCLAAPVK.V + Oxidation (M) |
|  |  |  | R.MICDCLAAPVK.V + 2 Carbamidomethyl (C) |
|  |  |  | R.IQTVLCDMLLR.D + Oxidation (M) |
|  |  |  | R.VRMICDCLAAPVK.V + Carbamidomethyl (C) |
|  |  |  | K.RLWGLVVCHHTSSR.F + Carbamidomethyl (C) |
|  |  |  | K.GQVWLLGITPTDEQIK.N |
|  |  |  | R.MICDCLAAPVKVIQDK.K + Carbamidomethyl (C); Oxidation (M) |
|  |  |  | R.VMVYKFHEDEHGEVISEYR.T |
|  |  |  |  |
| 39 | gi|18409228 | Ninja-family protein AFP1 | K.EMQTLRR.M |
|  |  |  | R.LSSVDMNMK.M + Oxidation (M) |
|  |  |  | K.SEMPCVFTK.G + Oxidation (M) |
|  |  |  | R.HIVVNTSSPSNLL.- |
|  |  |  | R.TTSLPAESEEEWR.K |
|  |  |  | R.GRPSSGLPRWSATANK.S |
|  |  |  | K.LVRSSSVVVTMPLFR.E + Oxidation (M) |
|  |  |  | K.VSTETVAGATRGTGLMR.T + Oxidation (M) |
|  |  |  | K.ASSDEARSLPSTTQPQQETTTKPTNR.L |
|  |  |  | R.YGSGEEVRIMCVCHGDFLSPADFVK.H |
|  |  |  |  |
| 43 | gi|302771345 | ABC transporter | -.MSTQRQR.A |
|  |  |  | R.VYYVASTLR.G |
|  |  |  | R.HDATIASGDYIRSLR.L |
|  |  |  | R.LMVSTSNAIGQLVLVYK.R + Oxidation (M) |
|  |  |  | K.FAVLDECTSAVSADGEEK.L |
|  |  |  | R.LTEQMASIEGVTGQSVISK.D + Oxidation (M) |
|  |  |  | R.EVLFIVIQTLLLYSRTR.L |
|  |  |  | K.YHTAVLYLDGSQSGYGWRYEVLK.A |
|  |  |  | K.FSVAISELYSYTFKPVLDIIVFTR.S |
|  |  |  | R.VAIPAAIVNSGLKYMQTIISLAFQQR.L |
|  |  |  | R.DLSFELLPGHSIIIMGPNGSGKSSIFR.V |
|  |  |  |  |
| 56 | gi|18391384 | SNARE-interacting protein KEULE | R.DLIPTK.L |
|  |  |  | R.LLYEMLR.S |
|  |  |  | K.TGSSKSTWK.V |
|  |  |  | R.HAHIADASER.L |
|  |  |  | R.IFVFIVGGATR.S |
|  |  |  | K.LAAGIWNCLAK.H |
|  |  |  | K.LNDLIREQGLR.E |
|  |  |  | R.AAKSLDASTMTTLR.D |
|  |  |  | K.NTPGGFTLKFDLHK.K |
|  |  |  | R.QPLPSMDAIYFIQPTK.E + Oxidation (M) |
|  |  |  | K.LSSDDMTAVNNMSLLGSAVDAK.K |
|  |  |  | R.TPTWAKPRGSDDGYSSDSVLR.H |
|  |  |  | K.LAKLSSDDMTAVNNMSLLGSAVDAK.K |
|  |  |  |  |
| 90 | gi|255080042 | Mitochondrial carrier family | K.GIGTTVTR.A |
|  |  |  | K.DGIAGLYK.G |
|  |  |  | R.QGPVGFFR.G |
|  |  |  | R.FGLYEPLK.V |
|  |  |  | K.EQIAAAASNVHVPK.V |
|  |  |  | R.GFSAAALREATYSSLR.F |
|  |  |  | R.EATYSSLRFGLYEPLK.V |
|  |  |  | K.QFPPPSMYQVASQVIAK.D |
|  |  |  | R.GEVPLGMMAQTSAIFRSEGMR.G + 2 Oxidation (M) |
|  |  |  | -.MASSGTLAGIDQTTKEQIAAAASNVHVPK.V + Oxidation (M) |
|  |  |  | R.TAMVQQQLAGMRGEVPLGMMAQTSAIFR.S + 2 Oxidation (M) |
|  |  |  |  |
| 91 | gi|308810769 | K^+^-channel ERG and related proteins, contain PAS/PAC sensor domain (ISS) | R.ARNEGR.L |
|  |  |  | R.SSRLADR.T |
|  |  |  | R.VAHPLLPVK.F |
|  |  |  | R.LLLDHGVEK.L |
|  |  |  | R.AESTLDAGGGR.E |
|  |  |  | R.AAEALGADASGK.G |
|  |  |  | R.DAFNAFPELR.E |
|  |  |  | R.QRPTTALMVSR.A |
|  |  |  | R.HGGPAAWAGAVQR.R |
|  |  |  | R.RAATSPGDDLLGR.L |
|  |  |  | K.SSFPAFLSPTPRK.A |
|  |  |  | R.AESTLDAGGGREESAEACAR.T |
|  |  |  | R.ELHDAGGRHGGPAAWAGAVQR.R |
|  |  |  | K.ATRAWTTDRPTPGSTATQASPAR.R |
|  |  |  | R.CLFLYFLLDSGLPHDGSPMEYARR.A + Oxidation (M) |
|  |  |  |  |
| **Protein with binding function or cofactor requirement and cellular components** | | |  |
| 15 | gi|460989 | beta tubulin | R.YLTASAMFR.G |
|  |  |  | K.LAVNLIPFPR.L |
|  |  |  | R.FPGQLNSDLR.K |
|  |  |  | R.VSEQFTAMFR.R |
|  |  |  | R.FPGQLNSDLRK.L |
|  |  |  | R.VSEQFTAMFRR.K |
|  |  |  | R.MMLTFSVFPSPK.V |
|  |  |  | R.LHFFMVGFAPLTSR.G |
|  |  |  | R.AVLMDLEPGTMDSVR.T |
|  |  |  | K.GHYTEGAELIDSVLDVVR.K |
|  |  |  | R.TGPYGQIFRPDNFVFGQSGAGNNWAK.G |
|  |  |  |  |
| 20 | gi|108864224 | Endonuclease III-like protein 1, putative | K.TEAVASMR.S |
|  |  |  | R.LGWVFREGTK.Q |
|  |  |  | K.TTTPEQTRMSLEK.W |
|  |  |  | K.TTTPEQTRMSLEK.W + Oxidation (M) |
|  |  |  | R.AALSSTSSSIAMATTRSSSSR.V |
|  |  |  | R.AALSSTSSSIAMATTRSSSSR.V + Oxidation (M) |
|  |  |  | R.FGGDIPDSLNELLALKGVGPK.M |
|  |  |  | -.MPLALLARAALSSTSSSIAMATTR.S |
|  |  |  | K.CDMCGINNICPSAFKESSSPNPK.Q + Oxidation (M) |
|  |  |  | R.FAVLISTMMSSQTKDEVTHAAVER.L |
|  |  |  | R.FAVLISTMMSSQTKDEVTHAAVER.L + Oxidation (M) |
|  |  |  |  |
| 38 | gi|226531021 | Lipid binding protein | R.CNLVDRPVGYK.C + Carbamidomethyl (C) |
|  |  |  | K.SGIKPEVAITIPK.R |
|  |  |  | K.KSGIKPEVAITIPK.R |
|  |  |  | K.QSPQCLCAVMLSKTAK.K |
|  |  |  | R.CNLVDRPVGYKCGDYTLP.- + Carbamidomethyl (C) |
|  |  |  | -.MKGLLLLVLALVASAACLVAVR.G + Oxidation (M) |
|  | | | |
| 41 | gi|255553540 | Protein binding protein, putative | R.VALQKK.A |
|  |  |  | R.DLQNAK.S |
|  |  |  | K.RVALQK.K |
|  |  |  | R.IEEFLK.D |
|  |  |  | K.INVTNSHR.S |
|  |  |  | K.KAAAAMVAAEDYAR.R + Oxidation (M) |
|  |  |  | K.AAAAMVAAEDYARR.F |
|  |  |  | R.WVHCSCDGISDEK.Y |
|  |  |  | K.YVDEVMVSDGERTSR.I |
|  |  |  | K.HTRCHSCGSSVPGNGLSVR.W |
|  |  |  | K.SLFNENEDITQAHQDSEMLEASWILK.K |
|  |  |  |  |
| 49 | gi|18401203 | Protein pleiotropic regulator PRL2 | R.VWDIR.T |
|  |  |  | K.TIKMWK.E |
|  |  |  | K.IWDVATGVLK.L |
|  |  |  | K.GEFCHNMLSLQR.D + Oxidation (M) |
|  |  |  | M.TMIALNREVETQSLK.K |
|  |  |  | R.DIINAVAVNEDGVMVTGGDK.G + Oxidation (M) |
|  |  |  | K.IWDVATGVLKLTLTGHIGQVR.G |
|  |  |  | R.DLNNTGNPGKSTAILPAPGSFSER.N |
|  |  |  | R.AETIVQPGSLESEAGIYAACYDQTGSR.L |
|  |  |  |  |
| 51 | gi|255541734 | Structural maintenance of chromosome 1 protein, putative | R.HERDK.A |
|  |  |  | K.ENANLK.I |
|  |  |  | K.EKILAK.L |
|  |  |  | R.ESAENR.G |
|  |  |  | K.TSMEMSK.I + 2 Oxidation (M) |
|  |  |  | R.LNQENGSLK.H |
|  |  |  | K.EKEDQISR.L |
|  |  |  | R.MINNNEIQK.S |
|  |  |  | K.NELEAALETSR.N |
|  |  |  | K.GAVDQSPNQQHK.S |
|  |  |  | K.EQLERNLALAR.E |
|  |  |  | K.NRLVAAMQATHELQIK.Q + Oxidation (M) |
|  |  |  | K.ASTNNTHVIKGAVDQSPNQQHK.S |
|  |  |  | K.NELEAALETSRNASPGETSLDGK.V |
|  |  |  |  |
| 60 | gi|164652942 | 14-3-3e protein | K.VFYLKMK.G |
|  |  |  | K.DSTLIMQLLR.D |
|  |  |  | K.SAQDIALAELAPTHPIR.L |
|  |  |  | K.LAEQAERYEEMVEFMEK.V |
|  |  |  | R.LGLALNFSVFYYEILNSPDR.A |
|  |  |  | R.LGLALNFSVFYYEILNSPDRACNLAK.Q |
|  |  |  | K.QAFDEAISELDTLGEESYKDSTLIMQLLR.D |
|  |  |  |  |
| 62 | gi|126508572 | 14-3-3 protein Lil 1433-3 | K.NEHER.K |
|  |  |  | K.AAGDDEH.- |
|  |  |  | K.EAADQSMK.A |
|  |  |  | K.MKGDYYR.Y |
|  |  |  | K.DSTLIMQLLR.D |
|  |  |  | R.LDLDLTVEER.N |
|  |  |  | R.YLAEFKNEHER.K |
|  |  |  | K.QAFDEAIAELDTLSEESYKDSTLIMQLLR.D |
|  |  |  |  |
| 78 | gi|148878501 | RecName: Full=Ribosome-inactivating protein PD-L3/PD-L4; | K.WIVLR.V |
|  |  |  | K.YATFMESLRNEAK.D + Oxidation (M) |
|  |  |  | K.LQDASSKTITLMLR.R + Oxidation (M) |
|  |  |  | K.CYGIPMLPDSNLTPK.Y + Oxidation (M) |
|  |  |  | K.TEAEFLLVAIQMVSEAAR.F + Oxidation (M) |
|  |  |  | -.VNTITFDVGNATINKYATFMESLR.N |
|  |  |  | R.SQVQLGIQILNSDIGKISGVSTFTDK.T |
|  |  |  |  |
| 85 | gi|11094250 | Cytosolic phosphoglucose isomerase | K.VLATQVRK.Q |
|  |  |  | R.DLMSDANR.C |
|  |  |  | K.SQQPVYLK.G |
|  |  |  | R.EWITAALGASAVAK.H |
|  |  |  | K.THLRDLMSDANR.C |
|  |  |  | K.FAPHIQQVSMESNGK.G + Oxidation (M) |
|  |  |  | -.MASSPALICDTEAWKDLK.G + Oxidation (M) |
|  |  |  | K.FGIDPNNAFAFWDWVGGR.Y |
|  |  |  | R.TQGTAPEGFNYSTTTLLKR.Y |
|  |  |  | R.MFNGEHINSTENRSVLHVALR.A + Oxidation (M) |
|  |  |  | K.DVIAIGIGGSFLGPLFVHTALQTDPEALESAK.G |
|  |  |  |  |
| 89 | gi|58013197 | Actin | K.AGFAGDDAPR.A |
|  |  |  | R.AVFPSIVGRPR.H |
|  |  |  | K.GEYDESGPSIVHR.K |
|  |  |  | K.IWHHTFYNELR.V |
|  |  |  | R.LDLAGRDLTDSLMK.I |
|  |  |  | K.NYELPDGQVITIGAER.F |
|  |  |  | K.YPIEHGIVSNWDDMEK.I |
|  |  |  | R.VAPEEHPVLLTEAPLNPK.A |
|  |  |  | -.MADGEDIQPLVCDNGTGMVK.A |
|  |  |  | R.CPEILFQPSLIGMEAPGIHETTYNSIMK.C |
|  |  |  |  |
| **Function unknown and hypothetical proteins** | | |  |
| 9 | gi|224092117 | Predicted protein | R.LSAKNR.E |
|  |  |  | K.SSVGTATVR.L |
|  |  |  | K.NMIPCSSPCR.Y + Oxidation (M) |
|  |  |  | R.ALYMTALEITK.S |
|  |  |  | R.LMVQDCNASSIK.S |
|  |  |  | K.LSLSIMNHEGVR.G + Oxidation (M) |
|  |  |  | R.WVSMAMSATTMITTYEFLKR.L |
|  |  |  | R.GLGPRWVSMAMSATTMITTYEFLK.R |
|  |  |  |  |
| 21 | gi|49388823 | Hypothetical protein | K.SVVGSTK.D |
|  |  |  | K.LAVAKNR.L |
|  |  |  | R.TGTGGSGLSGTCTCR.R |
|  |  |  | R.LAGMAAEISLLKSVVGSTK.D + Oxidation (M) |
|  |  |  | R.CSLHSEASSPSMPSSGLPSTDAAAEISTR.R |
|  |  |  | R.CSLHSEASSPSMPSSGLPSTDAAAEISTR.R + Oxidation (M) |
|  |  |  |  |
| 25 | gi|168005449 | Predicted protein | K.LKAQVR.E |
|  |  |  | R.DFWTAK.L |
|  |  |  | R.DKSGQNR.K |
|  |  |  | K.TELLVIEK.A |
|  |  |  | R.CETEKMR.S |
|  |  |  | K.SWDLEWEK.F |
|  |  |  | K.LVLEYEAWIKEK.E |
|  |  |  | K.MRSEIEVLQNEVSK.L |
|  |  |  | R.ELEEYNEDLSAQLR.K |
|  |  |  | K.VLFLPIGEMAVGTDESSDFSLGR.Y + Oxidation (M) |
|  |  |  |  |
| 52 | gi|18409257 | Uncharacterized protein | -.MGKWR.A |
|  |  |  | K.DDYAVK.E |
|  |  |  | K.VGLFER.S |
|  |  |  | R.YLDIVR.R |
|  |  |  | K.LFQLVGK.A |
|  |  |  | R.AVAALLLR.N |
|  |  |  | K.FIEHNIHFLQEVMQNR.Q + Oxidation (M) |
|  |  |  | R.YAQIYEYLREEYEISQR.F |
|  |  |  |  |
| 66 | gi|168040725 | Predicted protein | K.EADMKDK.G |
|  |  |  | R.DCKEADMK.D |
|  |  |  | R.QCGEETKGDR.R |
|  |  |  | K.EGWGLAVEEVLKNTEDLR.E |
|  |  |  | R.GGGNISSHHIGCGAGTTHPMR.T + Oxidation (M) |
|  |  |  | R.RGGGNISSHHIGCGAGTTHPMR.T |
|  |  |  |  |
| 67 | gi|115461348 | Os04g0678700 | K.SDYPSR.R |
|  |  |  | K.SDYPSRR.L |
|  |  |  | R.LMLDDLKK.S |
|  |  |  | R.LMLDDLKK.S + Oxidation (M) |
|  |  |  | K.WHVVMACR.D + Oxidation (M) |
|  |  |  | K.GFVSEAESGKR.L |
|  |  |  | K.ICNMLTMQEFHRR.F |
|  |  |  | R.AVATPAAPVASPGAGTSKADGK.K |
|  |  |  | -.MALQVQAALLPSALSVPKK.G + Oxidation (M) |
|  |  |  | K.TLRQGVVVITGASSGLGLAAAK.A |
|  |  |  | R.GQNGSAMIDGAESFDGAKAYK.D |
|  |  |  |  |
| 71 | gi|2058273 | YK426 | K.GYRYK.M |
|  |  |  | K.GTITEDA.- |
|  |  |  | K.LTRNFK.H |
|  |  |  | R.RTMAAIR.T + Oxidation (M) |
|  |  |  | K.VDMLEGVTILR.S + Oxidation (M) |
|  |  |  | K.HLNLDFQLLEGGRK.L |
|  |  |  | K.VKDELVLDGNDIELVSR.S |
|  |  |  | R.TAISHVQNLITGVTKGYR.Y |
|  |  |  | K.TILASETMEIPEGLTVQVAAKVVTVEGPR.G + Oxidation (M) |
|  |  |  |  |
| 72 | gi|293333271 | Hypothetical protein LOC100383295 | K.LGGVHIPK.G |
|  |  |  | R.AYSADVISR.T |
|  |  |  | K.NLTMVIQETLR.L |
|  |  |  | K.MKNLTMVIQETLR.L |
|  |  |  | K.LIVEPELGVDLTLTK.V |
|  |  |  | K.IDDDIRAYSADVISR.T |
|  |  |  | R.LYPAGAFVSRQALQELK.L |
|  |  |  | M.VDLMVDSAQPLLMSWEER.V + 2 Oxidation (M) |
|  |  |  | K.IRELQQAVSKPNVLAEMTGLR.F + Oxidation (M) |
|  |  |  | K.LSPHYQHSPTLKLIVEPELGVDLTLTK.V |
|  |  |  |  |
| 74 | gi|15239608 | Uncharacterized protein | K.DDGILR.L |
|  |  |  | R.TCLEKK.V |
|  |  |  | R.RTCLEK.K |
|  |  |  | K.LLCFRR.K |
|  |  |  | R.FIGGNSTFK.A |
|  |  |  | -.MSLLLNQPLK.L |
|  |  |  | K.VPLELVHSDPMVTIGSSHGWVATLK.D + Oxidation (M) |
|  |  |  | K.DDMFRIPGSGGHLIGSWDLGTHMHTPK.I + 2 Oxidation (M) |
|  |  |  |  |
| 77 | gi|224082162 | Predicted protein | R.DIYMYK.G + Oxidation (M) |
|  |  |  | R.RASADFLETPNFLR.A |
|  |  |  | K.FDLNTASSEAAPPSDHPQK.Q |
|  |  |  | K.QVGYGGMIDQRSSAATGSPR.T |
|  |  |  | K.QVGYGGMIDQRSSAATGSPR.T + Oxidation (M) |
|  |  |  | R.DIYMYKGDSAFCSQECR.Q + Oxidation (M) |
|  |  |  | R.TTSLTEIKFDLNTASSEAAPPSDHPQK.Q |
|  |  |  |  |
| 81 | gi|224094680 | Predicted protein | K.KAMVTK.E |
|  |  |  | K.AMVTKEK.G |
|  |  |  | K.DDVTEELK.I |
|  |  |  | K.EKGEIVLLCR.E |
|  |  |  | R.TSNILLDSTFR.A |
|  |  |  | R.TSNILLDSTFRAK.I |
|  |  |  | K.AIMEGTMDLHEHYK.I + 2 Oxidation (M) |
|  |  |  | K.IGGSVYRANINGCVLAVK.K |
|  |  |  | K.WMDPNLERFYPIDSAMSLATLAR.L |
|  |  |  | R.THLENGIEHLITYVWQPGDDLKK.V |
|  |  |  |  |
| 82 | gi|297832366 | Hypothetical protein ARALYDRAFT_343373 | K.VMNGIK.F + Oxidation (M) |
|  |  |  | R.EFFRR.K |
|  |  |  | K.MRVAQR.F + Oxidation (M) |
|  |  |  | R.YIAQRK.L |
|  |  |  | -.MLLASSR.Y + Oxidation (M) |
|  |  |  | K.VREICK.C |
|  |  |  | R.LIKYNR.Y |
|  |  |  | R.SLMSEMK.E |
|  |  |  | K.GLCLTNR.I |
|  |  |  | R.FFQEMER.K + Oxidation (M) |
|  |  |  | K.EDKVMNGIK.F + Oxidation (M) |
|  |  |  | K.VMEVLCNEGR.V + Oxidation (M) |
|  |  |  | -.MLLASSRYVPLTR.R + Oxidation (M) |
|  |  |  | K.VMNGIKFVEDMAER.G |
|  |  |  | R.WEDALEFLLKMEK.L + Oxidation (M) |
|  |  |  | R.LILDQKSASGALETFR.W |
|  |  |  | R.EICKCILTGLDYLHR.E |
|  |  |  | K.TDAIRWNFATFNTLIR.G |
|  |  |  | K.RPTAQQCLQHPWLNLR.T |
|  |  |  | K.EGNGYGEDEDHLALMMELLGKMPR.K + Oxidation (M) |
|  |  |  |  |
| 83 | gi|116790018 | Unknown | R.AERAGFK.A |
|  |  |  | R.FFQLYVYK.N |
|  |  |  | K.VDLSTTVLGFK.I |
|  |  |  | R.FRPRILIDVTK.V |
|  |  |  | K.NFEGLDLGKMEK.T |
|  |  |  | K.ISMPIMIAPTAMQK.M + Oxidation (M) |
|  |  |  | M.EIVNVSDYEVVAKQK.L |
|  |  |  | R.QLDYVPATISSLEEVVK.A |
|  |  |  | K.MVFDYYASGAEDQWTLHENR.K + Oxidation (M) |
|  |  |  | K.VDLSTTVLGFKISMPIMIAPTAMQK.M |
|  |  |  |  |
| 86 | gi|115456089 | Os03g0807800 | R.AGQRTR.F |
|  |  |  | R.GYWGNK.I |
|  |  |  | R.GSGIVAAR.V |
|  |  |  | M.AERGGER.G |
|  |  |  | K.TLGNFVK.A |
|  |  |  | R.MVPAPRGSGIVAAR.V |
|  |  |  | R.GAIILAKLSVVPVR.R |
|  |  |  | K.TYGFLTPDFWRDTK.F |
|  |  |  | K.KVLQFAGIEDVFTSSR.G |
|  |  |  | K.FVKSPFQEYTDLLAKPTK.A |
|  |  |  | K.IEEIYLHSLPVKEHQIVETLVPGLK.D |

^a^Assigned spot number as indicated in Figure 3 A and Table 2.

^b^Database accession numbers according to NCBInr.

^c^The name of the proteins identified by MALDI-TOF MS.
